# CircFAT1 Promotes Lung Adenocarcinoma Progression by Sequestering miR-7 from Repressing IRS2-ERK-mediated CCND1 Expression

**DOI:** 10.7150/ijbs.70889

**Published:** 2022-06-13

**Authors:** Hang Peng, Wan Zhang, Huanhuan Dong, Jialan Yuan, Yankun Li, Fanni Li, Daping Yu, Yingjie Guan, Feng Zhang

**Affiliations:** 1 Bone and Joint Research Center, The First Affiliated Hospital of Xi'an Jiaotong University, Xi'an, Shaanxi 710061, China.; 2 Department of Talent Highland, The First Affiliated Hospital of Xi'an Jiaotong University, Xi'an, Shaanxi 710061, China.; 3 Second Department of Thoracic Surgery, Beijing Chest Hospital, Capital Medical University, Beijing Tuberculosis and Thoracic Tumor Research Institute, Beijing 101149, China.; 4 Department of Medicine, Rhode Island Hospital, Providence, RI 02903, USA.

**Keywords:** circFAT1, miR-7-5p, IRS2, LUAD, DDP

## Abstract

Our understanding of coding gene functions in lung cancer leads to the development of multiple generations of targeted drugs. Noncoding RNAs, including circular RNAs (circRNAs), have been demonstrated to play a vital role in tumorigenesis. Uncovering the functions of circRNAs in tumorigenesis and their underlying regulatory mechanisms may shed new light on the development of novel diagnostic and therapeutic strategies for human cancer. Here we report the important role of circFAT1 in lung adenocarcinoma (LUAD) progression and the potential impact of circFAT1 on LUAD treatment. We found that circFAT1 was one of the top expressed circRNAs in A549 cells by circRNA-seq and was significantly upregulated in human LUAD tissues. Multiple cellular assays with A549 and PC9 LAUD cell lines under both gain-of-function and loss-of-function conditions demonstrated that circFAT1 promoted proliferation of LUAD cells *in vitro* and *in vivo*. At molecular level, circFAT1 sequestered miR-7 to upregulate IRS2, which in turn regulated downstream ERK1/2 phosphorylation and CCND1 expression, ultimately promoting tumor progression. In addition, we showed that DDP treatment was much more effective in circFAT1 knockdown tumor cells *in vitro* and in a xenograft tumor model. Our results indicate that circFAT1 promote tumorigenesis in LUAD through sequestering miR-7, consequently upregulating IRS2-ERK1/2-mediated CCND1 expression, and can be a valuable therapeutic target and an important parameter for precision treatment in LUAD patients.

## Introduction

Lung cancer is one kind of the malignant tumors with high morbidity and mortality worldwide 1. Non-small cell lung cancer (NSCLC), which accounts for 80%-85% of lung cancer with a low five-year survival rate of about 20%, is further divided into lung adenocarcinoma (LUAD), lung squamous carcinoma (LUSC) and large-cell lung cancer (LCLC) 2-4. In contrast, small cell lung cancer (SCLC) is less frequent with a five-year survival rate of 1%-5% 5, 6. LUAD is the most common type of NSCLC 7. The treatments of NSCLC include surgery, radiotherapy, chemotherapy, targeted therapy and emerging immunotherapy 8. Recent advances in molecular mechanisms of NSCLC lead to successful development of several generations of small molecule drugs specifically targeting certain genetic mutations of coding genes 9, 10, such as *EGFR*, *HER2*, *ALK*, *KRAS*, *BRAF*. Despite these progressions, the five-year survival rate for NSCLC remains low. Discovering novel molecular markers and drug targets, including non-coding RNAs, is essential for further improving the diagnosis and treatment of NSCLC, including LUAD.

Circular RNAs (circRNAs), in contrast to linear RNAs, are a group of looped single-stranded nucleic acid molecules, which are resistant to digestion by nucleic acid exonucleases and thus more stable in cells and body fluids, a property desirable for biomarkers of diseases and therapeutic drugs for delivery 11, 12. They are widely expressed, cell and tissue-specific and conserved across species 13. CircRNAs were initially thought to be byproducts of splicing during transcription, but it was found out later that circRNAs play important roles in a variety of cellular processes, including cell proliferation, migration, apoptosis, differentiation and autophagy 14-17. MiRNAs are another type of noncoding RNAs that bind untranslated region (UTR) of target RNAs to form an RNA-induced silencing complexes (RISCs) and repressing target gene expression 18. The majority of studies so far have shown that circRNAs are localized in the cytoplasmic compartment to act primarily as miRNA sponge by competitively sequestering specific miRNAs, and consequently releasing targeted mRNAs from RISCs 15, 17, 19, 20. It has also been reported that circRNAs can sequester protein or act as scaffolds to stabilize protein complexes 21, 22. In the nucleus, circRNAs, generally consisting of intron only or both introns and exons of the host genes, are able to interact with transcriptional complexes to regulate gene transcription, especially on their host genes 23. Although circRNA is categorized as non-coding RNA, it has been demonstrated that certain circRNAs contain m^6^A modification or internal ribosome entry sites (IRES) to initiate translation, and open reading frames (ORF) to encode functional polypeptides 24-26. CircRNAs can be dysregulated and contributed to pathological conditions, and therefore, could be developed as biomarkers for the diagnosis and therapeutical targets.

CircFAT1, also known as circFAT1(e2) and hsa_circ_0001461, is a product of 3,283 nucleotides in length, back-spliced from the exon 2 of the *FAT1* pre-mRNA. It has been reported to play an important role in tumorigenesis of multiple organ systems. In squamous cell carcinoma, circFAT1 regulates cancer stemness and antitumor immunity by binding to and activating STAT3 27. Studies in osteosarcoma, cervical cancer, hepatocellular carcinoma, and colorectal cancer indicate that circFAT1 regulates cell proliferation, invasion/migration and other cancer cell behavior through miR-375/YAP1, miR-409-3p/CDK8, miR-30a-5p/REEP3, miR-520b and 320c-3p/UHRF1 cascades 28-31. While circFAT1 functions as an oncogene in these systems, intriguingly, it is a tumor suppressor in gastric cancer by targeting miR-548g/RUNX1 in cytoplasm and physically interacting with YBX1 protein to block its function in nucleus 32. Additionally, *in vitro* studies suggested that circFAT1 could also regulate some cell biological properties by regulating miR-525-5p/SKA1, miR-181b/HK2, miR-30e-5p/ITGA6 or USP22, miR-873/ZEB1, miR-10a maturation, and miR-21 expression 33-39, however the *in vivo* importance and clinic relevance of circFAT1 and its downstream targets in tumorigenesis remain to be determined. These studies indicate that the same circRNA functions to regulate different miRNA targets in different systems, or by other completely different mechanisms.

In this study, we profiled circRNA expression in A549 cells. Initial functional screening and examination of circFAT1 expression in tumor tissues suggested an importance role of the circRNA in LUAD tumorigenesis. Both knockdown and overexpression of circFAT1 demonstrated that circFAT1 promoted cell proliferation. We also identified circFAT1 downstream regulatory cascades. In addition, circFAT1 expression level dramatically influenced effectiveness of cisplatin (DDP) treatment. Our study suggests that circFAT1 promotes LUAD tumorigenesis and is a promising therapeutic target and marker for precision treatment of LUAD.

## Materials and Methods

### Patient samples

A total of 34 pairs of LUAD and matched adjacent nontumorous tissues were obtained from patients who were diagnosed with LUAD and received surgery without a history of radiotherapy and/or chemotherapy at Beijing Chest Hospital (Beijing, China). All tissue specimens were frozen in liquid nitrogen after surgical resection and stored at -80 °C until RNA extraction. This study was reviewed and approved by the Medical Ethics Committee of Beijing Chest Hospital, and written informed consent was provided in accordance with the Declaration of Helsinki of the World Medical Association.

### Cell culture

Human LUAD cell lines (A549 and PC9) and HEK293T cells were cultured in RPMI 1640 (Hyclone, USA) or DMEM (Hyclone, USA) supplemented with 10% fetal bovine serum (FBS) (MRC, USA) and without antibiotics. All these cell lines were maintained at 37 °C with 5% CO_2_ in a humidified incubator.

### RNA isolation, cDNA synthesis and RT-qPCR

Total RNA was extracted with TRIzol reagent (Takara, Dalian, China) and reverse-transcribed into cDNA with PrimeScript RT Reagent Kit (Applied Biological Materials, Canada) as described by the manufacturers. RT-qPCR was performed using Agilent AriaMx Real-Time PCR System (Agilent, USA) with PowerUP^TM^ SYBR^TM^ Master Mix (Life Technologies, USA). *GAPDH* was used as internal reference for quantification of circRNA and mRNA, and *U6* snRNA was used for miRNA. The relative expression levels of genes were calculated by 2 ^-ΔΔCT^ method. The specific primers for all PCR reactions are listed in Additional file: Table S1, S2.

### RNase R treatment assay

2 μg of total RNA was digested with 3 U/μg RNase R (Epicenter, WI, USA) for 15 min at 37 °C followed by incubation at 80 °C for 5 min to stop the reaction. RNase R-treated RNA and -untreated control RNA was reverse-transcribed separately, and qPCR was performed to quantify the relative levels of circRNAs and linear RNAs of their host genes.

### CircRNA and mRNA sequencing

For circRNA-seq, total RNA was treated with RiboZero rRNA Removal Kit (Epicentre, WI, USA) to deplete ribosomal RNA (rRNA) following the manufacturer's protocols. The rRNA-depleted RNA samples were digested by RNase R to remove linear RNA followed by purification using Agencourt RNAClean XP magnetic beads. For mRNA-seq, total RNA was processed to enrich mRNAs with poly(A) tails by Oligo (dT) magnetic beads. The enriched circular RNA or mRNA samples were randomly fragmented into small pieces. DNA Libraries were prepared using TruSeq® Stranded kit and sequenced with HiSeq2500 (Illumina, San Diego, USA).

### Plasmid construction, lentivirus packaging and cell transfection/transduction

The full-length cDNAs of circRNAs were amplified by PCR from an A549 cDNA library and cloned into pLCDH-ciR overexpression vector (Geneseed, China), which contains a front and back circular frame to facilitate cyclization of circRNAs. The empty vector without any insert served as negative control. To knock down circRNAs, the corresponding DNA sequences of designed shRNAs against the junction sites of circRNAs (shcircRNAs) and negative control shRNA (shNC) against luciferase 40 were synthesized and cloned into pLKO.1 vector. All of the cloned insert sequences were verified by Sanger sequencing. The miRNA mimic and inhibitor were purchased from RiboBio (Guangzhou, China). Cell transfection was performed with Lipofectamine 3000 (Invitrogen, Carlsbad, CA, USA) in accordance with the manufacturer's protocol. For the gain-of-function and loss-of-function assays of circFAT1 and IRS2, lentiviruses were first packaged in HEK293T cells by co-transfection of the cloned lentivirus vectors with pVsvg, pGag/Pol and pRev plasmids, then supernatant was collected, and lentiviruses were concentrated by PEG6000 precipitation. The cells were transduced by lentiviruses in the presence of 8 μg/mL of polybrene and selected with 2 μg/mL puromycin to obtain stably transduced cells. The sequences of shRNAs and the primers for circRNAs cloning are listed in Additional file: Table S3.

### CCK8, BrdU, colony formation, cell cycle and apoptosis assays

Cell viability of A549 and PC9 cells harboring shcircFAT1, shNC, pLCDH-circFAT1 or pLCDH-ciR transgenes were detected by Cell Counting Kit-8 (CCK8) as manufacturer described (InCellGenELLC, USA). For BrdU assay, cells were seeded into 48-well plates (10^4^ cells per well) for 2 days, and replaced with complete culture medium containing 3 μg/mL BrdU and continued to incubate for 2 h. The labeled cells were fixed with 4% paraformaldehyde for 1 h, and treated with 2N HCl at 37 °C for 30 min, followed by neutralization with sodium tetraborate. The treated cells were then stained with anti-BrdU antibody (1:500, Santa Cruz Biotechnology, USA) at 4 °C overnight. After three washings, the cells were incubated with Cy3-conjugated secondary antibodies (1:300, Proteintech, China) at room temperature for 1 h, washed again, stained the nuclei with DAPI and observed under a fluorescence microscope (Leica, Germany). For cell colony formation assay, cells were seeded into 6-well plates (5×10^2^ cells per well) for 7-10 days. After washing twice with PBS and fixing with 4% paraformaldehyde for 30 min, cells were stained with 0.1% crystal violet solution for 30 min. Colonies with more than 50 cells were counted under a microscope. Cell cycle analysis was implemented by flow cytometry. Briefly, cells were harvested and fixed with 70% ethanol at 4 °C for 2 h. After washing once with PBS, the cells were stained with propidium iodide (PI) solution supplemented with RNase A at 37 °C in the dark for 30 min. The percentage of cells in each cell cycle stage was detected by a flow cytometer (Becton, Dickinson and Company, USA). For apoptosis assay, cells were harvested after transduction with shcircFAT1 or shNC lentiviruses for 3 days, and double-stained with fluorescein isothiocyanate (FITC)-conjugated Annexin V and 7-amino-actinomycin D (7-AAD). The early and late apoptotic cells were analyzed on a flow cytometer. All experiments were repeated in triplicate.

### Isolation of nuclear and cytoplasmic fractions

Nuclear and cytoplasm fractions were separated as previous described 41. Briefly, cells were rinsed twice with DEPC-treated PBS, and lysed in 300 μL of 0.3% NP40-containing NIB-250 buffer (15 mM Tris-HCl pH 7.5, 60 mM KCl, 15mM NaCl, 5 mM MgCl_2_, 1 mM CaCl_2_ and 250 mM sucrose) supplemented with protease and RNase inhibitors on ice for 15 min. The cell lysates were transferred into 1.5 mL centrifuge tubes and centrifuge at 510 g at 4 °C for 10 min. The supernatants were collected as cytoplasmic lysates and the pellets were washed once with NIB-250 buffer as nuclei. RNA was extracted by TRIzol method according to the instruction.

### Immunofluorescence assay (IF)

Immunofluorescence assay was performed as previous described 42. Briefly, tumor tissue was fixed in 4% paraformaldehyde for 24 h following by dehydration with 30% sucrose and frozen in O.C.T. Compound (Fisher Scientific, USA), cryosectioned at 8 μm, and mounted on slides. Tissue sections were permeabilized with PBS containing 0.3% Triton X-100 and blocked in PBS containing 1% BSA at 4 °C for 1 h, and then incubated with primary antibodies in PBS at 4 °C overnight, followed by three washings in PBS and further incubation with Cy3-conjugated secondary antibodies (1:300, Proteintech, China) at room temperature for 1 h and by another three washings. The primary antibodies used were anti-IRS2 (1:100, Proteintech, China), anti-CCND1 (1:100, Servicebio, China), anti-Ki67 (1:500, Proteintech, China). Sections were stained with DAPI and mounted using antifade mounting medium (VECTASHIELD, USA), photographed with a fluorescence microscope.

### Dual-luciferase reporter assay

The DNA fragments containing the putative miR-7 binding sites in circFAT1 and IRS2 3'UTR and their corresponding deletional mutants were PCR amplified, cloned into luciferase reporter vector pmiR-Glo (Promega, USA), and named as pcircFAT1-3'UTR-WT, pcircFAT1-3'UTR-Mut, pIRS2-3'UTR-WT and pIRS2-3'UTR-Mut. The primer pairs used in PCR reaction are listed in Additional file: Table S3. All cloned sequences were verified by Sanger sequencing. The luciferase activity was performed with a Dual-Luciferase Reporter Assay system (Promega, USA) in accordance with the manufacturer's protocols.

### RNA pulldown assay

RNA pulldown assay was performed as described 43. Briefly, biotin-labeled circFAT1-specific probe (5'-ACTGTCGGGAATCTGTCTCTTCACC-3'-Biotin) and control probe (5'-AGATCACCAAGAGGTGCAACATTAG-3'-Biotin) were synthesized by Sangon Biotech (Shanghai, China). A549 or PC9 cells were fixed with 1% paraformaldehyde for 15 min., and 1.25% glycine was utilized to terminate fixation, then the cells were collected after washing with PBS and lysed with 500 μL of lysis buffer (50 mM Tris HCl pH 7.0, 10 mM EDTA, 1% SDS, 200 U/mL of RNase inhibitor, 5 μL/mL of proteases inhibitor cocktail and PMSF) at 4 ℃, sonicated for 30 s followed by centrifuging at 12,000 g at 4 ℃ for 10 min and the supernatants were collected for hybridization. Two volumes of hybridization buffer (50 mM Tris-HCl pH 7.0, 750 mM NaCl, 1 mM EDTA, 1% SDS and 15% Formamide) were added to the supernatants, and 5% volumes of the mixture were transferred into new centrifuge tube as input. The remaining mixture was divided into two tubes to incubate with 100 nM circFAT1 specific probe or control probe at 20 °C overnight, and then incubated with streptavidin-conjugated magnetic beads with rotation at room temperature for additional 2 h to pull down hybridized RNA complexes. The magnetic beads were washed 5 times with 2× sodium citrate buffer, digested with protein K at 50 °C for 45 min and de-crosslinked at 95 °C for 10 min. The magnetic beads were discarded and the supernatants were collected for RNA isolation with TRIzol reagent. The abundance of circFAT1 and miR-7 were detected by RT-qPCR.

### Western blot

Cells were lysed with RIPA buffer (Beyotime, China) supplemented with 1 mM phenylmethylsulfonyl fluoride (PMSF) for 10 min on ice and centrifuged at 14,000 × g. The supernatants were collected, and whole cell extracts were separated by 10% SDS-PAGE gel and transferred onto a PVDF membrane (GE, USA). The membranes were blocked with 5% skimmed milk (BD, USA) in TBST buffer and incubated with primary antibodies at 4 °C overnight. Primary antibodies used were anti-IRS2 (1:1,000, Proteintech), anti-CCND1 (1:1,000, Servicebio, China), anti-ERK1/2 (1:1,000, CST, USA), anti-p-ERK1/2 (1:1,000, Proteintech, China), and anti-β-actin (1:1,000, Sigma, USA). After washing with PBS five times, the membranes were incubated with near-infrared fluorescent anti-mouse (1:50,000, LI-COR, USA) or anti-rabbit (1:25,000, Jackson, USA) secondary antibodies for 1 h at room temperature. Fluorescent signal was detected by Odyssey Near-infrared fluorescence imaging system (LI-COR, USA).

### Northern blot

10 μg of total RNA or RNase R treated total RNA was denatured in a buffer containing 6.6% formaldehyde and then electrophoresed in a formaldehyde agarose gel at a voltage of 5 V/cm. The RNA was then capillary transferred to a positively charged nylon membrane in an alkaline transfer solution (0.01 M NaOH, and 3M NaCl). The membrane was prehybridized in prehybridization solution (5×SSC, 50% formamide, 10×Denhardt, 0.01M sodium phosphate buffer (pH 6.6), 0.5% SDS, 40 μg/mL denatured salmon sperm DNA) at 42 °C for 2 h. Subsequently, biotin-labeled circFAT1-specific probe (5'-ACTGTCGGGAATCTGTCTCTTCACC-3'-Biotin) and FAT exon 2 probe (5'-ACTCAGGCTCATGACAGTAGTACCA-3'-Biotin) were added to the prehybridization solution and hybridized overnight at room temperature. HRP-coupled streptavidin was used to detect biotin.

### Mouse xenograft model

All animal care and procedures were conducted according to the guidelines of the National Institutes of Health and approved by Xi'an Jiaotong University Animal Care and Use Committee. The stable cell lines with circFAT1 knockdown or overexpression were established by transducing corresponding lentiviruses into A549 cells and selected with puromycin. Cells transduced with lentivirus bearing shNC without circFAT1 insertion were used as controls. For xenograft experiments, 1×10^7^ modified A549 cells were subcutaneously inoculated into female BALB/c nude mice for five mice per group. The volume of tumors was measured every other day and calculated as 0.5×length×width^2^. The mice were sacrificed after 4 weeks, and the tumors tissues were collected and weighed. For DDP sensitivity assay, DDP was injected intraperitoneally, starting from 12 days after cells inoculation, three times a week for 3 weeks. The dose of DDP were 9 mg/kg/week or 14 mg/kg/week. Equal volume of PBS was injected in the control group. The volume of tumors was measured every two days and calculated as 0.5×length×width^2^. The mice were sacrificed after three weeks, and the tumor tissues were weighed and collected for RT-qPCR and immunostaining analyses.

### Statistical analysis

Statistical analyses were performed with GraphPad Prism 7.0 (GraphPad Software Inc., CA, USA). The differences between groups were assessed by Student's t test, one-way ANOVA. Data were showed as mean ± standard deviation (SD). *P* value < 0.05 was considered as statistically significant.

## Results

### CircRNA profiling of A549 human LUAD cells

**Figure 1:**
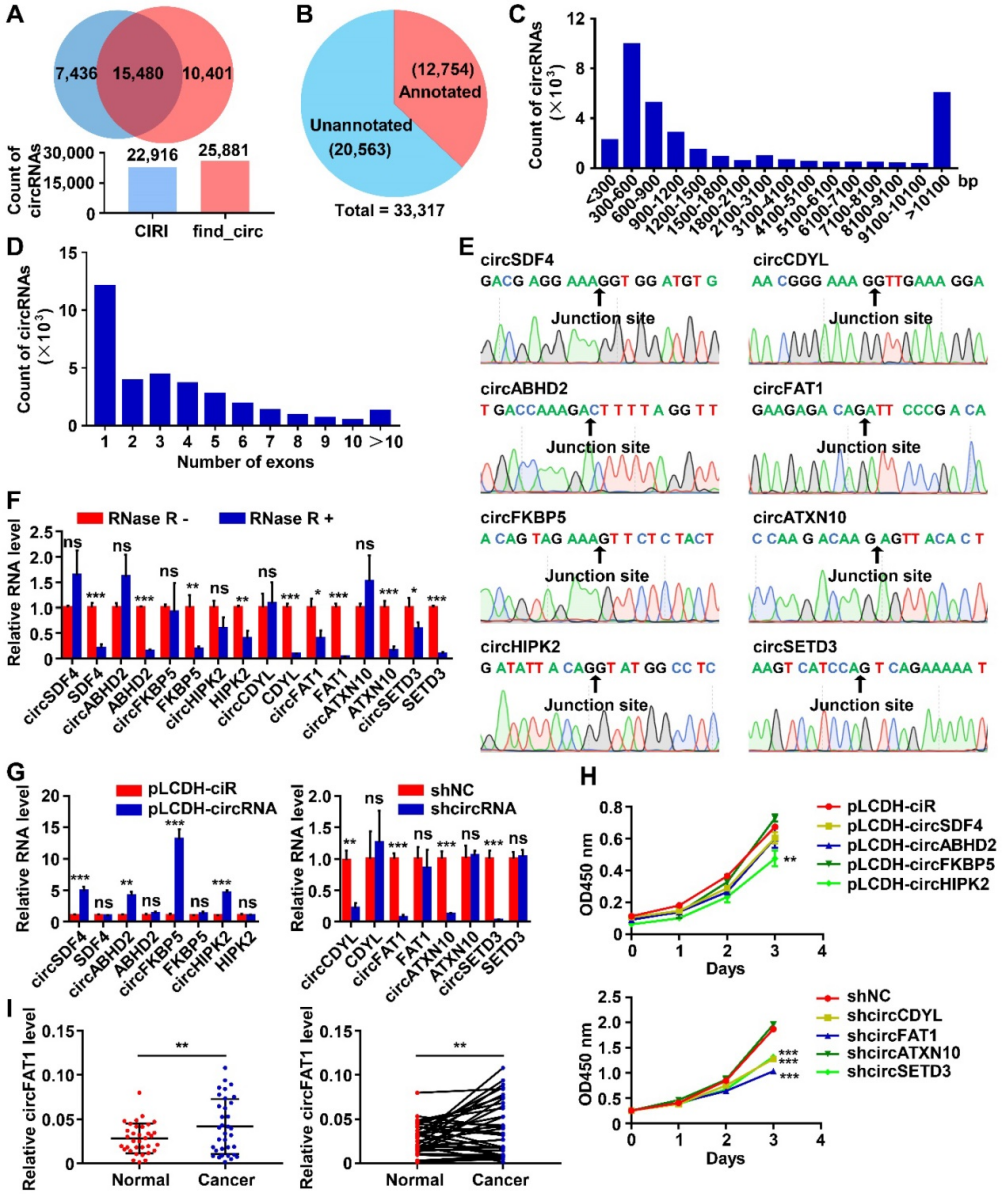
** CircRNA profiling in A549 LUAD cells and functional screening of circRNAs. (A)** The numbers of circRNAs identified by CIRI and find_circ from A549 circRNA-seq data. (Upper) Venn diagram showing the numbers of circRNAs identified by CIRI only (light blue), both CIRI and find_circ, and find_circ only (orange); (Lower) The total numbers of circRNA identified by each of the algorithms as indicated. **(B)** Pie chart showing the numbers of identified circRNAs annotated and unannotated by circBase. **(C** and** D)** The distribution of lengths (C) and exon numbers (D) of the identified circRNAs. bp, base pairs. **(E)** Sanger sequencing confirmed the sequences of 8 circRNAs at backsplicing junction sites. **(F)** RT-qPCR analyses demonstrating that the indicated circRNAs were more resistant to RNase R digestion than their linear RNA counterparts. **(G)** RT-qPCR analysis to show the efficiency of circRNA overexpression (Left, pLCDH-circRNAs; pLCDH-ciR for control) or knockdown (Right, shcircRNA; shNC for control) in A549 cells by transducing with corresponding lentiviruses. **(H)** CCK8 assay to screen circRNAs promoting A549 cell growth. The indicated vectors were used to produce lentiviruses for transducing transgenes into A549 cells. (Upper) circRNA overexpression, (Lower) circRNA knockdown. **(I)** RT-qPCR assay to analyze relative expression of circFAT1 in paired LUAD tissues and adjacent paracancerous tissues from 34 patients, showing circFAT1 was expressed significantly higher in tumor tissues. GAPDH were used for normalization. *P < 0.05, **P < 0.01, ***P < 0.001, ns, not significant.

To survey circRNAs expressed in lung cancer cells, we performed circRNA-seq, which removes linear RNAs before library construction and specifically detects circRNAs, in one of the most commonly used lung cancer cell lines, A549 cells. All circRNAs indentified by two algorithms, CIRI 44 and find_circ 45, were combined, resulting in a total of 33,317 circRNAs (Table S4), and 15,480 of them were detected by both of the tools (Figure 1A). Among them, 12,754 circRNAs were already annotated in circBase, accounting for 38.3% of all circRNAs that we identified, and the majority of the identified circRNAs (61.7%) were unannotated (Figure 1B). These data demonstrated the sensitivity of the profiling method that we employed and the complexity, potentially, the functional importance of circRNAs in a cellular system. The circRNA host genes were distributed throughout all chromosomes as shown by Circos plot 46 (Figure S1A). 96.9% of circRNAs in A549 cells (32,283 out of 33,317) were located in annotated intragenic regions, while only 1,034 of them (3.1%) were in intergenic regions (Figure S1B), indicating that the majority of circRNAs were the circularized splicing products of annotated linear transcripts. The length of circRNAs was mainly between 200 bp-1,500 bp (64.6% of the total, Figure 1C), and 36.2% of intragenic circRNAs were formed by a single annotated exon (Figure 1D).

To validate the identified circRNAs, we first used divergent and convergent primer pairs, which detect circular form only and both circular and linear form, respectively (Figure S1C). All of the tested circRNAs were detected from an A549 cDNA library by PCR assay with the expected sizes of the PCR products, but not from human genomic DNA (gDNA) when divergent primers were used, whereas convergent primer pairs amplified PCR product from both the cDNA and gDNA as expected (Figure S1D). The precise sequences flanking the back-splicing junction sites of the circRNAs were verified by Sanger sequencing of the RT-PCR products (Figure 1E). Furthermore, RNase R digestion, which degrades linear RNAs, of testing total RNA dramatically reduced linear RNA PCR signals amplified by linear RNA-specific primer pairs (Figure 1F and S1C) with virtually no effects on the PCR products from circRNAs. These data confirmed the presence of all circRNAs that we tested.

### Screening of candidate circRNAs important for the growth of A549 cells

To identify circRNAs important for tumorigenesis in lung cancers, we estimated the roles of circRNAs in the growth of A549 cells by CCK8 assays, which detects dehydrogenase activities, a marker of total cell viability. Lentiviruses expressing medium level of the candidate circRNAs or high level of shRNAs specifically against the back-splicing junction sequence of the candidate circRNAs were constructed to perform gain-of-function or loss-of-function assays (Figure S1E-F). In our initial screening, a total of 8 circRNAs with successful overexpression or effective knockdown without affecting the expression of corresponding linear transcript levels were selected for further studies (Figure 1G). Among overexpressed circRNAs, circHIPK2 inhibited the proliferation of A549 cells, while circSDF4, circABHO2 or circFKBP5 had no significant effects (Figure 1H). Knockdown assay suggested that circCDYL, circFAT1 or circSETD3, but not circATXN10 possibly inhibited the proliferation of A549 cells (Figure 1H). To evaluate the relevance of the circRNAs to lung cancer, we examined cancer tissues and paraneoplastic tissues from 34 LUAD patients by the RT-qPCR assay and found that the expression level of circFAT1 in cancer tissues were significantly higher than that in paraneoplastic tissues (Figure 1I, S1G), suggesting that circFAT1 may contribute to the development and/or progression of lung cancers, and thus circFAT1 was selected to further explore its function and the underlying mechanisms in LUAD. The presence of circFTA1 in A549 cells was also confirmed by Northern blot with two biotin-labeled single-stranded oligonucleotide DNA probes. The circFAT1-specific probe targeting the back-splicing junction of circFAT1 detected a single band in A549 cells and the signal was reduced upon knockdown of circFAT1, and the probe targeting the exon 2 region of FAT1 detected two transcripts: circFAT1 and linear FAT1 mRNA as expected (Figure S1H).

### CircFAT1 promotes LUAD cells proliferation *in vitro*

**Figure 2:**
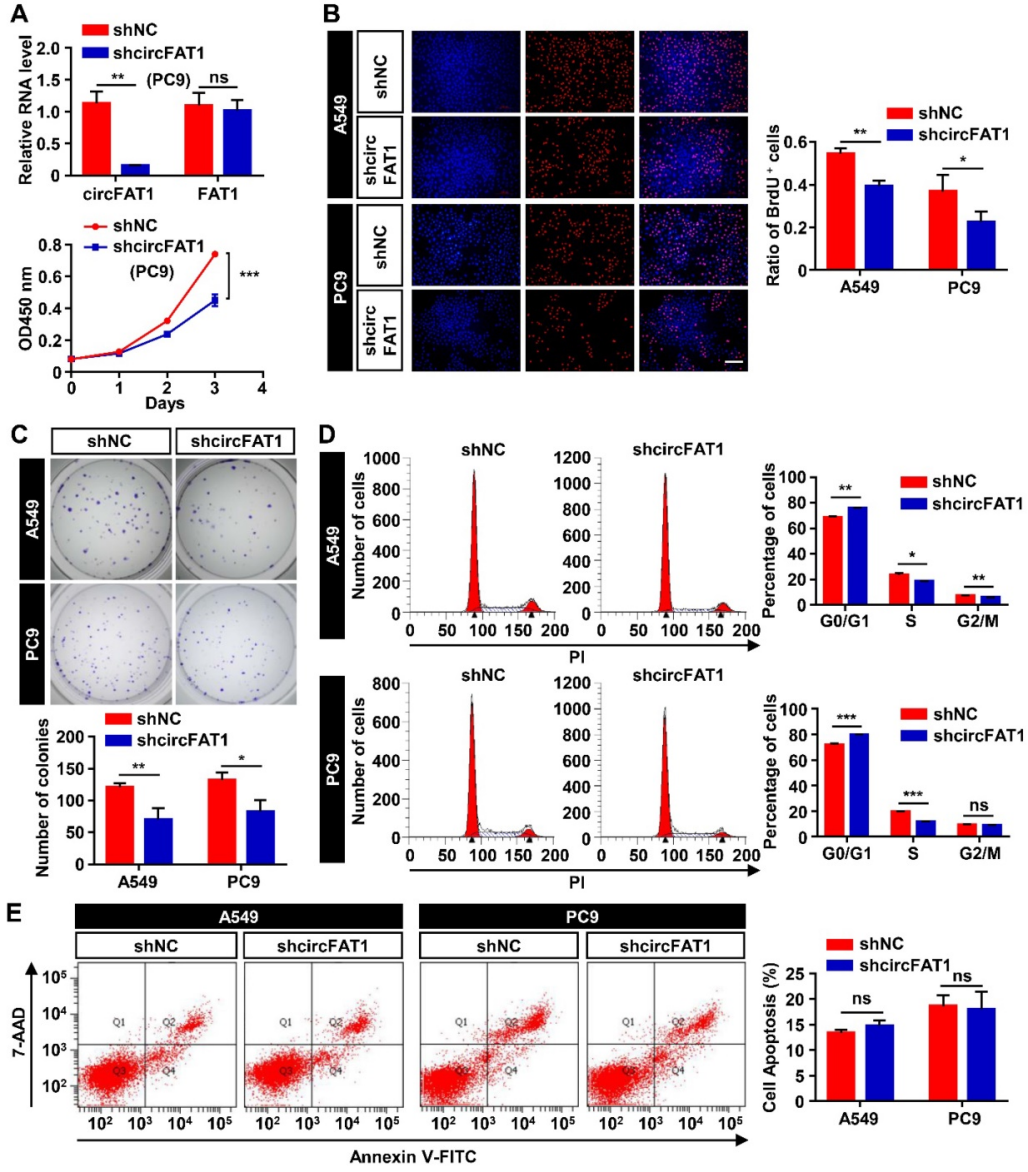
** CircFAT1 promotes A549 and PC9 LUAD cells proliferation (**Loss-of-function assays; for gain-of-function assays, see Fig. S2)**. (A)** (Upper) RT-qPCR analysis to show knockdown efficiency of shcircFAT1 in PC9 cells. (Lower) CCK8 assays to show the growth curves of PC9 cells bearing shcircFAT1 or shNC. **(B)** BrdU incorporation assay showing reduction of BrdU labeled cells in shcircFAT1-expressing cells after two-hours exposure to BrdU (Left) and quantification of BrdU labeled cells (Right). **(C)** Colony formation assay to evaluate clonogenicity of shcircFAT1 cells (Upper) and quantification of colonies with more than 50 cells (Lower). Cells were stained with crystal violet. **(D)** shcircFAT1 increased cells at G0/G1 phase and decrease cells at S phase in both A549 and PC9 cells. (Left) Representative flow cytometry data of cells stained with PI, (Right) quantification of cells at different cell cycle stages. **(E)** Apoptosis rate was analyzed by flow cytometry analysis of cells stained with FITC-conjugated Annexin V and 7-AAD (left) and quantification of apoptotic cells. The assay was performed three days after transduction of shcircFAT1 lentivirus. Data are collected from triplicate and shown as mean ± SD, *P < 0.05, **P < 0.01, ***P < 0.001, ns, not significant.

Initial screening of circRNAs in A549 cells suggested circFAT1 enhanced cell viability and likely promoted cell proliferation. To ascertain the role of circFAT1 in cell proliferation and to further define cellular functions of circFAT1, we performed multiple cell biological assays using two different human LUAD cell lines, A549 and PC9 cells. Stable cell populations were generated from the two cell lines by lentivirus transduction of circFAT1 or shcircFAT1 genes, followed by puromycin selection to eliminate cells without successful integration of transgenes into the genomes. The most effective shcircFAT1 knocked down circFAT1 by 85.4% in PC9 cells, and the shRNA had no effect on the expression of FAT1 mRNA as demonstrated in A549 cells (Figure 1G, 2A); similarly, stable overexpression of circFAT1 enhanced the circRNA expression, without significantly altering FAT1 mRNA levels in both A549 and PC9 cells (Figure S2A). These reagents, therefore, are suitable for our functional studies. We first performed the CCK8 assays, revealing that downregulation of circFAT1 significantly decreased the cell viability in both A549 and PC9 cells (Figure 1H, 2A), and that upregulation of circFAT1 significantly increased cell growth (Figure S2B). To confirm that circFAT1 regulates cell proliferation, we carried out BrdU assays, revealing that knockdown of circFAT1 markedly decreased the rate of BrdU incorporation (Figure 2B), and overexpression of circFAT1 displayed the opposite effect in both the cell lines (Figure S2C). Colony formation assay also showed that the cell colonization capabilities of A549 and PC9 were significantly impaired by downregulation of circFAT1 and markedly enhanced by upregulation of circFAT1 (Figure 2C, S2D). To further define the stages that circFAT1 regulates cell proliferation in LUAD cells, flow cytometry was employed to measure DNA content stained by propidium iodide (PI), showing that downregulation of circFAT1 resulted in an increase of cells at G0/G1 phase and a decrease at S phase (Figure 2D), and that upregulation of circFAT1 led to the opposite effect (Figure S2E). Additionally, flow cytometry analyses of cells stained with annexin V and 7-AAD, which is an indicator of early and late stage of apoptosis, demonstrated that downregulation of circFAT1 by shcircFAT1 lentiviruses for 3 days had no effect on apoptosis in both A549 and PC9 cells (Figure 2E). Together, these data clearly demonstrate that circFAT1 enhances the proliferation of the LUAD cells, but no effect on apoptosis.

### CircFAT1 functions as a sponge for miR-7

**Figure 3:**
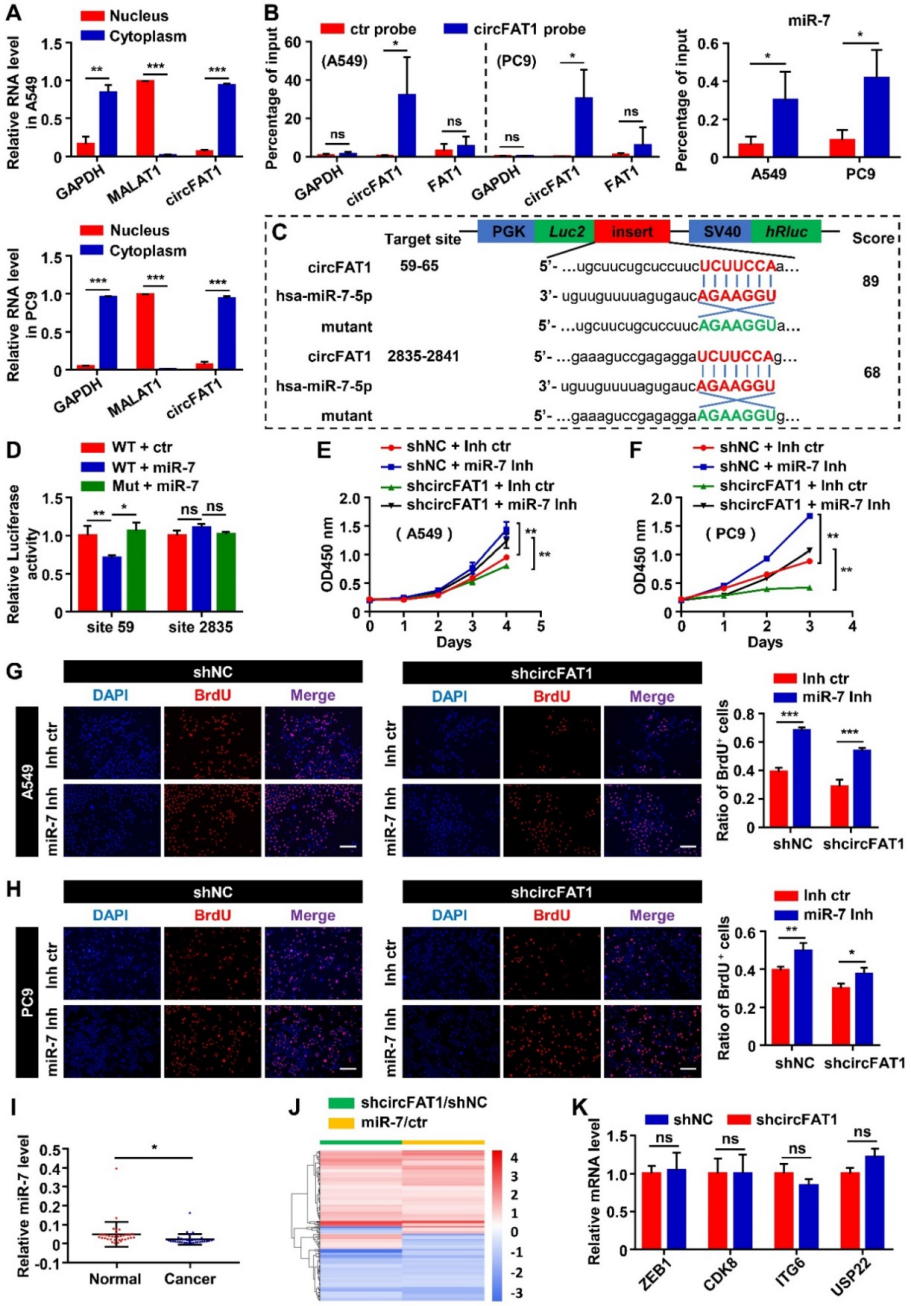
** CircFAT1 functions as a sponge for miR-7. (A)** RT-qPCR analysis of nuclear and cytoplasmic fractionations to detect the subcellular localization of circFAT1 in A549 (Upper) and PC9 cells (Lower). GAPDH and MALAT1 were used as cytoplasmic and nucleic RNA control, respectively. **(B)** circFAT1 and miR-7 were co-pulled-down by a circFAT1-specific probe from fixed A549 and PC9 cells. (Left) The circFAT1-specific probe drastically enriched circFAT1 but not GAPDH and FAT1 mRNA as detected by RT-qPCR. The control probe pulled-down background levels of the RNAs. (Right) MiR-7 was also enriched in the pull-down products by the circFAT1-specific probe over that of the control. **(C)** Schematic illustration of the luciferase reporter, the cloned circFAT1-WT and circFAT1-Mut miR-7-binding sequences, and hsa-miR-7-5p sequence. The scores of miR-7-binding sites predicted by CircInteractome were listed on the right side. **(D)** The relative luciferase activities in 293T cells after co-treatment of circFAT1-WT or circFAT1-Mut with miR-7 mimic or miRNA mimic control to show site 59 was capable of downregulating luciferase activity in the presence of miR-7 mimic. **(E-H)** Cell proliferation rescue assay showing that miR-7 inhibitor rescue shcircFAT1 phenotype analyzed in A549 and PC9 cells by CCK assays (E, A549; F, PC9) and by BrdU assays (G, A549; H, PC9; scale bar, 200 μm). **(I)** Relative expression of miR-7 detected by RT-qPCR in 34 paired LUAD tissues compared with adjacent normal tissues. **(J)** mRNA expression profiles of A549 cells after knockdown of circFAT1 or transfection with miR-7 mimic; heatmap showing that the genes co-regulated by both the RNAs were largely regulated in the same directions. **(K)** Relative mRNA expression levels of the reported circFAT1-associated miRNA targets in circFAT1 knockdown A549 cells. Data are presented as mean ± SD; *P < 0.05, **P < 0.01, ***P < 0.001, ns, not significant. ctr, miR-7 mimic control; miR-7, miR-7 mimic; miR-7 Inh, miR-7 inhibitor; Inh ctr, miR-7 inhibitor control.

CircFAT1 is a highly expressed circRNA in A549 cells (Listed top 9 by circRNA-seq, Table S4). To elucidate the molecular mechanism underlying the cellular function of circFAT1, we first performed RNA subcellular fractionation assay. RT-qPCR analysis of circFAT1 RNA from cytoplasmic and nuclear lysates revealed that virtually all of circFAT1 was presented in the cytoplasmic compartment of A549 and PC9 cells, in a similar fashion to cytoplasmic localized GAPDH mRNA, while nuclear localized MALAT1 noncoding RNA was detected only in nuclear extracts, which served as a control of the fractionation assay (Figure 3A). Thus, circFAT1, as most cytoplasmic localized circRNAs, might function as a miRNA sponge. To explore this possibility, potential miRNA targets of circFAT1 were predicted using CircInteractome database 47. The candidates with score above 85 were further evaluated by analysis of the candidate miRNA levels upon silencing circFAT1 in A549 cells. Most of tested candidate miRNAs were not expressed, decreased or had no significant change in circFAT1 knockdown A549 cells, including miR-375, miR-409-3p, miR-548g, miR-570, miR-1245, miR-296-5p, miR-192-5p, miR-602, miR-579-3p, miR-215, miR-892b, miR-1229-3p, miR-942, miR-619-3p, miR-1299, and miR-654-5p, but only miR-7 was increased (Figure S3A). Although circRNA-sponging doesn't necessarily reduce the targeted cellular microRNA levels, most reports demonstrated the reduction of microRNA levels by circRNA-sponging. Therefore, we first tested whether circFAT1 served as a ceRNA for miR-7.

We next performed circRNA pull-down assay with a biotin-labeled oligonucleotide probe, that specifically hybridized to the junction sites of circFAT1, to examine whether miR-7 was co-pulled-down with the circRNA. Both circFAT1 and miR-7 were enriched in the pull-down products of the circFAT1-specific probe over that of a mutated control probe (Figure 3B), indicating that miR-7 was capable of binding to the circRNA in the fixed cells. To further confirm sequence-specific interaction of the two RNAs, dual-luciferase reporter assay was carried out in HEK293T cells. Two miR-7 target sites of circFAT1 at nucleotide position of 59-65 and 2,835-2,841 were predicted by TargetScan (Figure 3C). A total of four DNA fragments flanking the predicted two miR-7-binding sites (circFAT1-WT site 59 and site 2,835) and their corresponding mutants (circFAT1-Mut site 59 and site 2,835) were cloned separately into the 3'UTR of the luciferase reporter in pmiR-Glo vector (Figure 3C). This analysis revealed that co-transfection of miR-7 mimic with the reporter harboring either miR-7-binding site 2,835 or its mutant significantly modified luciferase activity. In contrast, miR-7 mimic decreased the luciferase activity of the site 59-containing reporter, but not of the mutant reporter (Figure 3D). Collectively, these results demonstrate that miR-7 binds to circFAT1 in a sequence-specific manner, and thus, circFAT1 could serve as a competing endogenous RNA (ceRNA) of miR-7.

MiR-7 has been demonstrated as a tumor suppressor in several type of neoplasia 48. To investigate the functional consequences of miR-7 sequestration by circFAT1, we first examined the role of miR-7 in cell proliferation in our cellular systems. The CCK8 assay revealed that miR-7 mimic inhibited cell viability, and that miR-7 inhibitor enhanced cell viability (Figure S3B). BrdU assays also showed that the ratio of BrdU-positive cells in both of the cell lines that transfected with miR-7 mimic was lower than that transfected with the control, while the miR-7 inhibitor increased incorporation of BrdU into the cells, further supporting miR-7 inhibited proliferation of the two LUAD cell lines (Figure S3C). Next, we executed rescue experiment to determine whether miR-7 could reverse the function of circFAT1. MiR-7 inhibitor rescued the proliferation-suppressing effects of circFAT1 knockdown in both of the cell lines, whereas the miR-7 mimic diminished proliferation-promoting effects induced by overexpression of circFAT1 as detected by CCK8 and BrdU assays (Figure 3E-H, S3D-F). Importantly, we also found significantly lower levels of miR-7 in cancer samples than that in paired paraneoplastic samples from the 34 LUAD patients by RT-qPCR, which is consistent with the idea that circFAT1 downregulates miR-7 level and its function (Figure S3A, 3I). Together, these data demonstrate that circFAT1 functions as a miRNA sponge to achieve its proliferation-promoting effect.

To evaluate the importance of miR-7 in mediating the function of circFAT1 in our system, we analyzed genes co-regulated by the circRNA and the miRNA at genome-wide level. mRNA profiling of A549 cells with knockdown of circFAT1 or transfection of miR-7 mimic revealed expressional changes of 759 genes (Table S5) and 1756 genes (Table S6), respectively. Among 233 genes co-regulated by circFAT1 knockdown and miR-7 mimic, 197 genes were regulated in the same direction, accounting for 84.5% of the co-regulated genes (Figure 3J, Table S7), indicating that circFAT1- and miR-7-regulated programs were highly shared. This is consistent with the idea that they are in the same genetic pathway and demonstrates the importance of miR-7 in mediating circFAT1 function.

Multiple microRNAs were reported to mediate circFAT1 function in different systems along the way of the study, we therefore were interested in interrogating the role of those factors in A549 cells. Our small RNA-sequencing data (Table S8) and RT-qPCR analysis showed that miR-525-5p, miR-30a-5p, miR-375, miR-181b, miR-302c-3p, miR-520b and miR-548g were not expressed in A549 cells (Figure S3A). Although miR-873, miR-10a, miR-409-3p, miR-30e-5p were expressed, the expression levels of these microRNAs were downregulated or unchanged upon circFAT1 knockdown in A549 cells (Figure S3A), rather than being upregulated as shown in other systems 29, 35, 37, 38. Furthermore, we examined the expression level of mRNAs targeted by the expressed miRNAs, including ZEB1, CDK8, ITGA6 and USP22, expression levels were not significantly changed in circFAT1 knockdown cells (Figure 3K). Taken together, these data indicate that the reported circFAT1-regulated miRNAs and mRNAs are either unexpressed in A549 cells or expressed but being regulated in different manner, suggesting that circFAT1 exert its function through different strategies in different systems.

### IRS2 is a direct target of miR-7, and circFAT1 regulates LUAD cells proliferation via miR-7/IRS2/p-ERK1/2/CCND1 pathway

**Figure 4:**
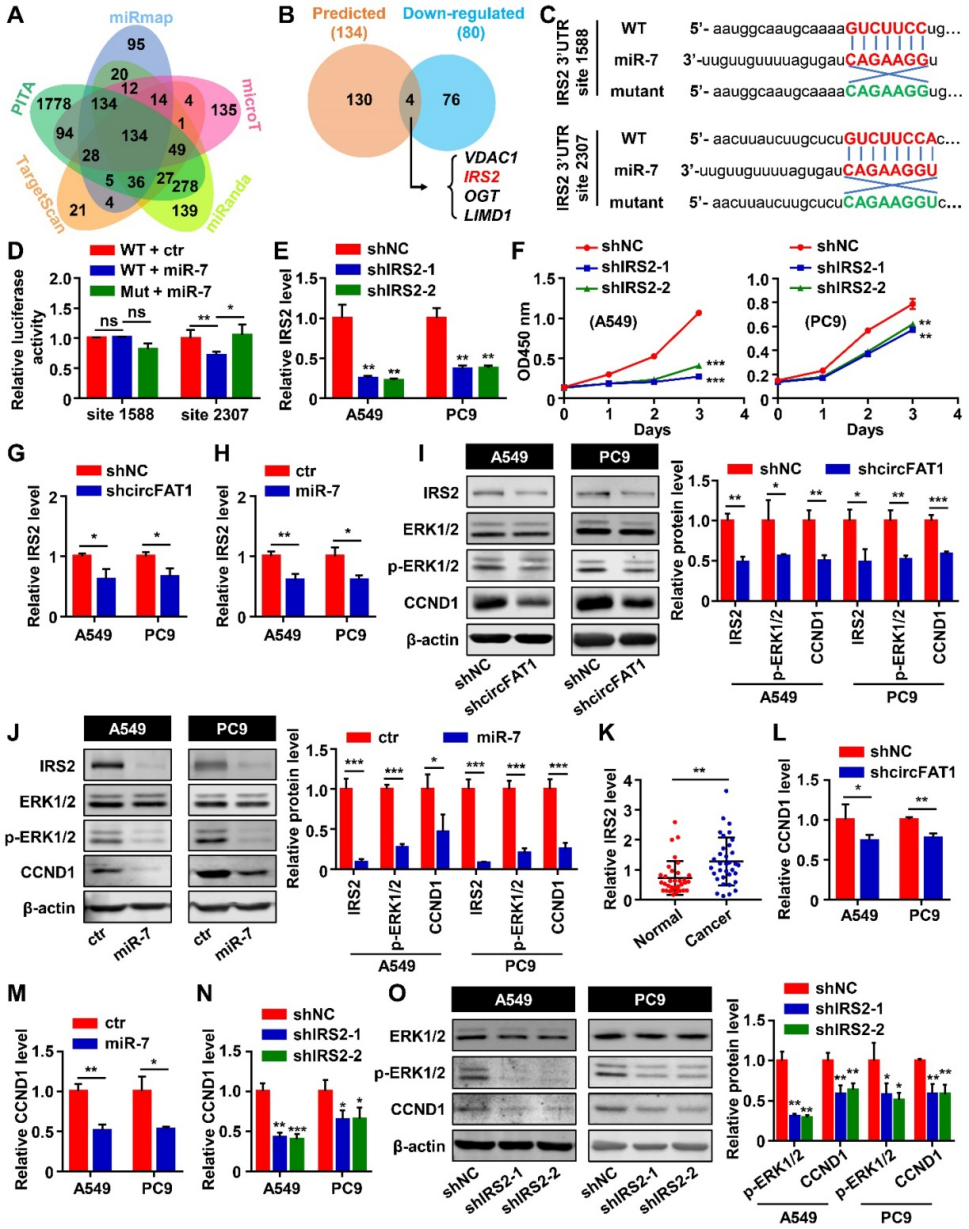
** IRS2 is a direct target of miR-7, and circFAT1 regulates LUAD cells proliferation via miR-7/IRS2/p-ERK1/2/CCND1 pathway. (A)** Potential target genes of miR-7 predicted by 5 different algorithms. The numbers of genes predicted by individual tools uniquely or different combinations were indicated. **(B)** Venn diagram showing the numbers of miR-7 target genes predicted by all five tools and downregulated by both miR-7 mimic and shcircFAT1. **(C)** Schematic illustration of two predicted miR-7-binding sites (sites 1588 and 2307) in IRS2 3'UTR and the sequences of the sites (3'UTR-WT) and their mutant (3'UTR-Mut) that cloned into luciferase reporter. MiR-7 sequence was included to show matched sequences (Red). The complementary sequences were used in mutants (Green). **(D)** The relative luciferase activities in 293 T cells after transfected with IRS2 3'UTR-WT or IRS2 3'UTR-Mut reporters and miR-7 mimic or mimic control to show site 2307 was functional. **(E)** Knockdown efficiencies of shIRS2 tested in A549 and PC9 cells as detected by RT-qPCR. **(F)** CCK8 assay showing growth curves of A549 and PC9 cells infected with shIRS2 or shNC. **(G)** Relative IRS2 mRNA expression level in A549 and PC9 cells transduced with shcircFAT1 as detected by RT-qPCR to show downregulation of IRS2 by shcircFAT1. **(H)** Relative mRNA expression level of IRS2 in A549 and PC9 cells transfected with miR-7 as detected by RT-qPCR, showing downregulation of IRS2 by miR-7. **(I and J, Left)** Western blot assay showing that shcircFAT1 (I) and miR-7 (J) downregulated IRS2, p-ERK1/2, CCND1, but not ERK1/2 in A549 and PC9 cells. (Right) Quantification of western blot signals. **(K)** Expression level of IRS2 in 34 paired LUAD clinical samples tested by RT-qPCR. **(L-N)** RT-qPCR assay showing downregulation of CCND1 mRNA in A549 and PC9 cells by shcircFAT1(L), miR-7 (M) and shIRS2 (N). **(O, left)** Western blot analysis to show that shIRS2 downregulated p-ERK1/2 and CCND1, but not ERK1/2 protein levels in A549 and PC9 cells. (Right) Quantification of western blot signals. Data are presented as mean ± SD; *P < 0.05, **P < 0.01, ***P < 0.001; ns, not significant; ctr, miR-7 mimic control; Inh, miR-7 inhibitor; Inh ctr, miR-7 inhibitor control.

We have demonstrated that circFAT1 exerts its biological function through sponging miR-7. To continue to uncover the downstream regulatory targets of circFAT1, we utilized starBase 49 to predict target genes of miR-7, resulting in 134 putative miR-7 targets predicted by all of the five miRNA prediction algorithms that we employed, namely TargetScan, PITA, miRmap, MicroT and miRanda (Figure 4A). Intersection of these predicted targets with 80 genes downregulated by both miR-7 mimic and shcircFAT1 treatments narrowed the candidates to 4 genes (Figure 4B), of which *IRS2* was a gene highly related to proliferation. Therefore, we focused on the possibility that IRS2 directly mediated miR-7 function in the A549 cells. Two binding sites of miR-7 were predicted in the 3'UTR of IRS2 through the miRDB 50, 51. We cloned DNA fragments containing the miR-7 binding sites of IRS2 and their corresponding mutants into 3'UTR of luciferase gene in pmiR-Glo reporter (Figure 4C). Dual-luciferase assays with these constructs showed that miR-7 mimic did not inhibit luciferase activity with the predicted site at nucleotide 1,588 cloned into the 3'UTR, neither did the mutant of the site, suggesting that site 1,588 wasn't functional (Figure 4D). However, miR-7 mimic inhibited the luciferase activity with site 2,307 cloned to its 3'UTR, and the inhibitory activity of miR-7 mimic was abolished with the mutation at site 2,307 (Figure 4D), indicating that miR-7 directly target IRS2 mRNA through miR-7-binding site 2,307. It has been reported that IRS2 regulates the cell proliferation in LUAD cells 52, 53. To ensure that IRS2 are capable of promoting the proliferation of A549 and PC9 cells, we again constructed two shRNAs against *IRS2* (shIRS2-1 and shIRS2-2) to knock down *IRS2* using lentiviral vector. RT-qPCR assays showed that the two shRNAs knocked-down IRS2 mRNA levels by 74.9% and 77.6% in A549 cells, 63.7% and 62.1% in PC9 cells. Similarly, the protein levels of IRS2 were reduced by 66.3% and 66.5% in A549 cells, 50.8% and 47.8% in PC9 cells (Figure 4E, S4A). CCK8 assay revealed that *IRS2* knockdown inhibited the proliferation of shIRS2-transduced A549 and PC9 cells, and BrdU assays likewise showed reduction of BrdU positive cells after the treatment (Figure 4F, S4B-C), confirming that IRS2 promoted cell proliferation in the LUAD cell lines. To confirm that circFAT1 and miR-7 are capable of regulating IRS2, we examined the mRNA levels and protein levels of IRS2 after manipulating circFAT1 or miR-7 levels. CircFAT1 knockdown or miR-7 mimic transfection downregulated the expression of IRS2 mRNA and protein levels in both of the cell lines (Figure 4I-J). Additionally, the expression levels of IRS2 in cancer tissue from the 34 LUAD patients were significantly higher than that in paired paraneoplastic tissue, supporting the idea that *IRS2* promote proliferation in LUAD (Figure 4K). These data demonstrate that IRS2 promote cell proliferation, and mediate circFAT1 and miR-7 function in the LUAD cell lines.

In mammary tumorigenesis, IRS1/2 regulates the phosphorylation of ERK1/2 and upregulates CCND1 54, 55. We found that the phosphorylation level of ERK1/2 and the protein level of CCND1 decreased after knocking down circFAT1 or transfecting miR-7 mimic in both the cell lines (Figure 4I-J). CCND1 mRNA levels in the treated cells were also decreased (Figure 4L-M). CCND1 regulates G1/S transition 56, which is consistent with the observations that downregulation of circFAT1 reduced the ratio of G0/G1 phase cells (Figure 2D and S2E). Therefore, circFAT1/miR-7 regulate LUAD cells proliferation via ERK1/2/CCND1 pathway. To investigate whether IRS2 are capable of regulating CCND1 via p-ERK1/2, we tested the CCND1 mRNA expression level in the cell lines after transducing shIRS2-1 or shIRS2-2 lentiviruses, and confirmed that downregulation of IRS2 inhibited CCND1 mRNA expression (Figure 4N) and attenuated the phosphorylation level of ERK1/2 as well as the protein levels of CCND1 (Figure 4O). In addition, A549 or PC9 cells treated with PD98059, an inhibitor of MEK, which is an upstream activator of ERK, also exhibited inhibition in proliferation capacity (Figure S4D) and decreased the rate of BrdU-positive cells (Figure S4E), as well as the downregulation of CCND1 in both mRNA level and protein level (Figure S4F-G). Together, these results demonstrate that circFAT1 regulate LUAD cell proliferation via miR-7/IRS2/ERK1/2/CCND1 cascade.

### CircFAT1 promotes tumor progression in a xenograft model

**Figure 5:**
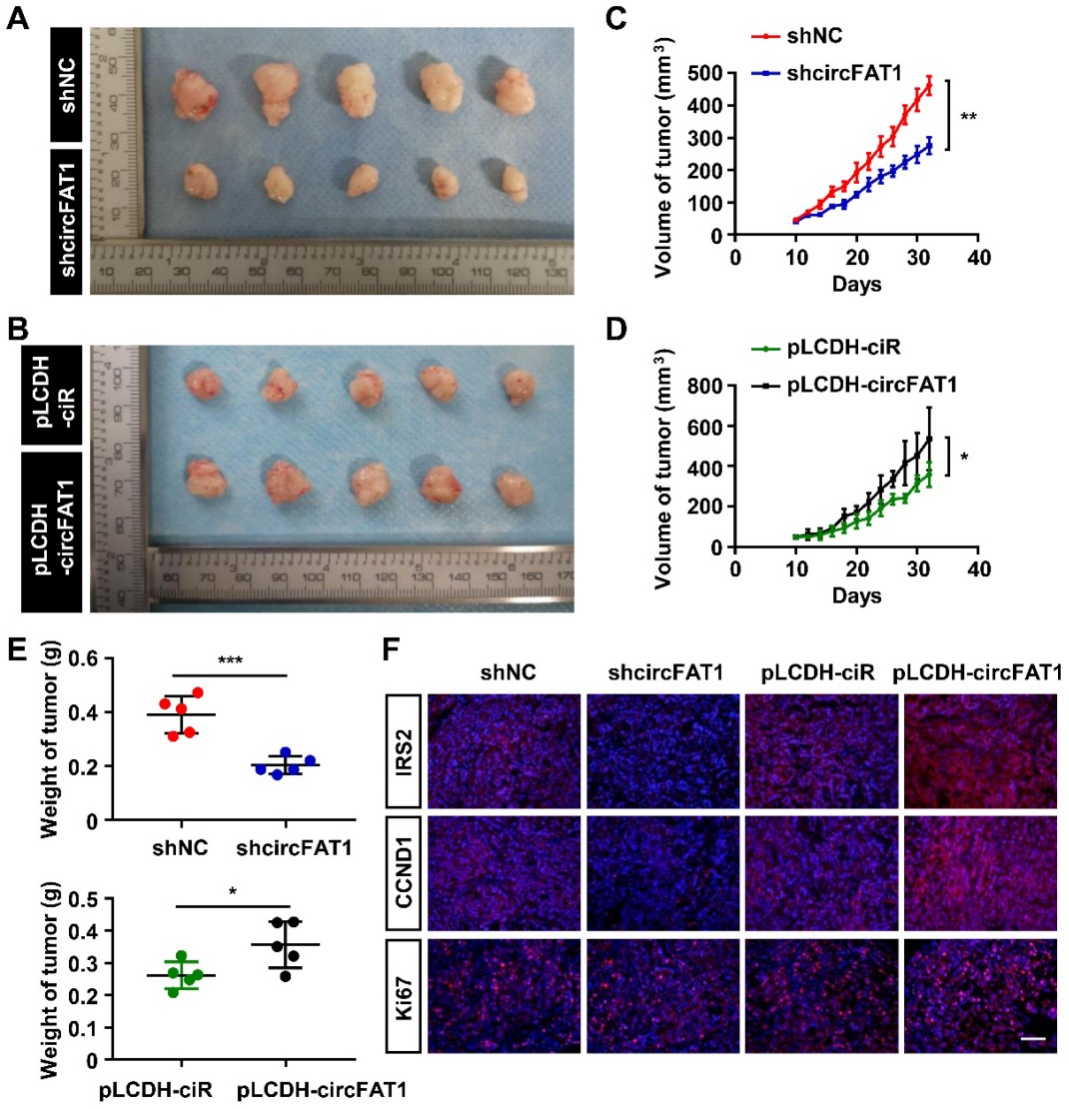
** CircFAT1 promotes A549 LUAD cell tumorigenesis *in vivo*. (A** and** B)** Xenograft assay showing A549 cells with circFAT1 knockdown (A) or overexpression (B) by lentiviruses inhibited or promoted tumor formation, respectively (n=5). **(C** and** D)** Volume of the xenograft tumors under circFAT1 knockdown (C) and overexpression (D) conditions. The volumes of tumors were estimated by measuring sizes every other day. **(E)** Weight of harvested xenograft tumors derived from A549 cells with circFAT1-knockdown (Upper) or overexpression (Lower). **(F)** Immunofluorescent staining of xenograft tumors showing that the protein levels of IRS2, CCND1 and Ki67 were decreased in circFAT1-knocked-down tumor tissues and increased in circFAT1-overexpressed tissue. Scale bar, 100 µm. Data are presented as mean ± SD; *P < 0.05, **P < 0.01, ***P < 0.001.

To examine the roles of circFAT1 in tumorigenesis *in vivo*, A549 cells stably expressing shcircFAT1 or shNC were subcutaneously injected into nude mice, and we found that both the size and weight of the tumors in shcircFAT1 group were reduced by 40.4% and 48%, respectively. Similarly, the tumors in circFAT1-overexpressing group were increased 1.5 fold in sizes and 1.4 fold by weight over tumors in control group (Figure 5A-E, S5A-B). The body weights of treated mice weren't significant altered in both circFAT1 knockdown and overexpression cases (Figure S5C-D). In addition, we also found that Ki67, IRS2 and CCND1 protein were upregulated in tumor tissues by an immune-fluorescence assay, which is consistent with the observations *in vitro* (Figure 5F). These results demonstrate a proliferation-promoting effect of circFAT1 on LUAD progression *in vivo*.

### Knockdown of circFAT1 further reduces tumor mass in DDP-treated mice in a xenograft model

**Figure 6:**
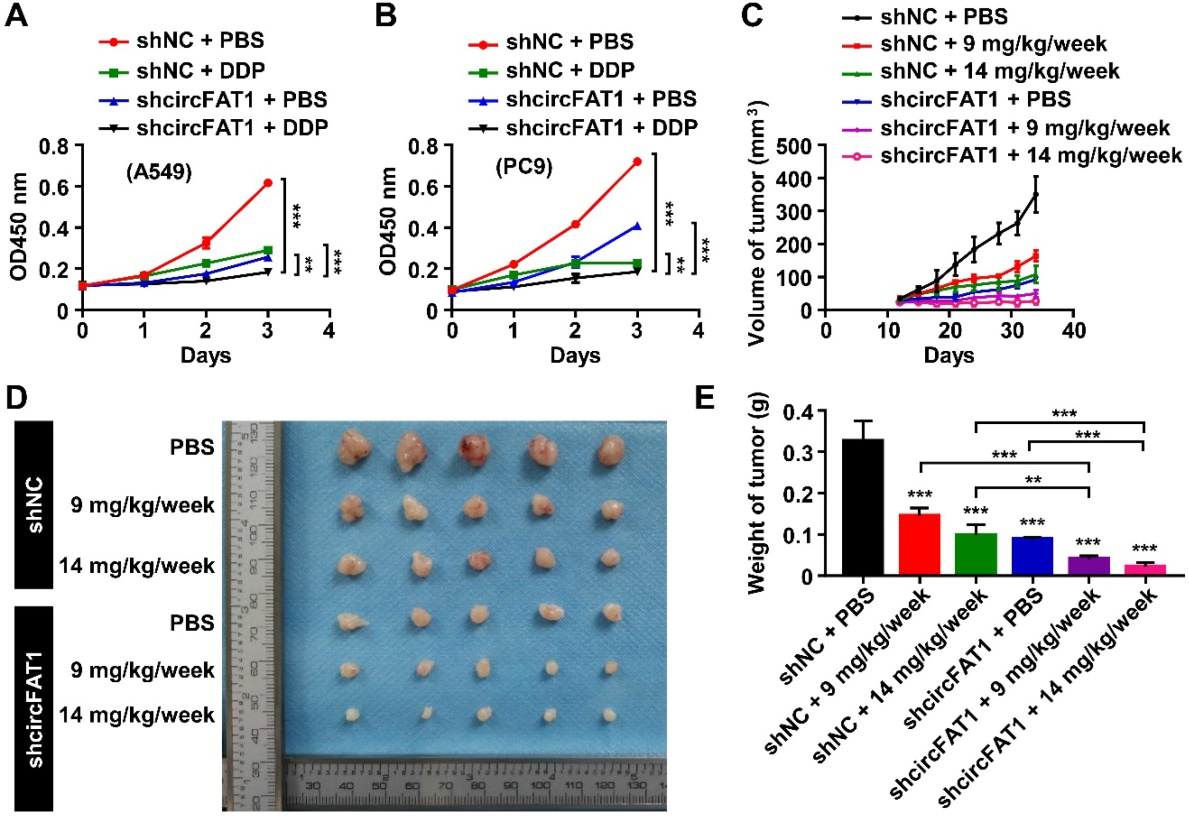
** Knockdown of circFAT1 further reduces the tumor size of DDP-treated mice*.* (A** and** B)** Growth curves of A549 (A) and PC9 (B) cells with circFAT1 knockdown and/or DDP treatment as evaluated by CCK8 assay. **(C)** Growth curves of xenograft tumors estimated by measuring the sizes every three days during 33 days of experimental period. **(D)** Xenograft tumors derived from A549 cells treated with shcircFAT1 or shNC and different dosages of DDP (n=5). **(E)** The weight of dissected xenograft tumors shown in (D) to demonstrate that knockdown of circFAT1 enhanced the effectiveness of DDP, and that 9 mg/kg/week of DDP with circFAT1 knockdown reduced tumor mass more than that of higher dosage of DDP treatment (14 mg/kg/week) without circFAT1 knockdown (shNC). Data in (A, B and E) are presented as mean ± SD, *P < 0.05, **P < 0.01, ***P < 0.001.

To investigate the therapeutical implications of circFAT1, we interrogated whether circFAT1 expression had any impact on lung cancer chemotherapy. DDP is one of the commonly used chemotherapy drugs. As expected, both DDP treatment and knockdown of circFAT1 alone showed inhibitory effect on the growth of A549 and PC9 cells by CCK8 assay, and the combination of both displayed significantly stronger inhibition on cell growth than either one alone (Figure 6A-B). We then asked whether tumors with lower circFAT1 expression response better to DDP treatment in a xenograft assay with BALB/C nude mice. A549 cells stably expressing shcircFAT1 and control cells were seeded into 15 nude mice each group, and two concentrations of DDP (9 and 14 mg/kg/week) and PBS control were intraperitoneal injected in 5 mice each sub-group for three times a week (Figure S6A). The volume and weight of the tumors from combined treatments of circFAT1 knockdown and DDP were remarkably smaller and lighter than circFAT1 knockdown or DDP treatment alone (Figure 6C-E, S6B). The body weights of DDP-treated mice were substantially reduced with time due to the cytotoxic side effects of the drug, while reduced circFAT1 in tumors essentially had no effect on mouse body weights during experimental period (Figure S6C). Furthermore, the average tumor weight of lower dosage of DDP treatment (9 mg/kg/week) combined with circFAT1 knockdown was less than half weight of high dosage of DDP treatment (14 mg/kg/week) alone (40.4 mg versus 98.5 mg). These results demonstrate that combined treatment of DDP and circFAT1 knockdown is more effective in the mouse model, suggesting that patients with lower level circFAT1 in tumors may response better to DDP treatment, and that lower dosage of DDP can be used in patients with lower circFAT1 expression in tumors to achieve comparable effects, which is highly beneficial to patients with severe side-effects to DDP treatment.

## Discussion

CircRNAs play important roles in physiological processes and diseases. Besides presence in tissues, they are also detected in body fluids, including blood, urine, saliva 57. Due to their high stability, circRNAs are suitable to be developed as diagnostic and prognostic markers, and therapeutic targets as well 58. In an effort to identify circRNAs important for the development of lung cancer, we profiled circRNAs in human A549 LUAD cells. Our initial mini-screening led us to focus on circFAT1 (Figure 1). Multiple cellular assays, including CCK8, BrdU labeling, colony formation and cell cycle analysis by flow cytometry indicated that, in both gain-of-function and loss-of-function conditions, circFAT1 promoted cell proliferation in two different kinds of adenocarcinoma cells that we tested. The role of circFAT1 in promoting tumorigenesis was further supported *in vivo* by a mouse xenograft assay (Figure 5). Analysis of clinical samples also confirmed that circFAT1 was upregulated in LUAD cancer tissues over paracancerous tissues. Our study demonstrates that circFAT1 promotes LUAD tumorigenesis.

We dedicated extensive efforts to unravel the molecular mechanism underlying circFAT1 function. Our results lead us to propose circFAT1 sponge miR-7 to downregulate IRS2 mRNA and protein levels, which, in turn, reduce phosphorylation of ERK1/2 and CCND1 mRNA and protein levels. The importance of miR-7 in mediate circFAT1 function was further supported by the fact that most co-regulated genes by the two RNAs were regulated in parallel. Additionally, we found that circFAT1 knockdown caused G0/G1 phase arrest (Figure 2F) is consistent with CCND1 is a regulator of G1/S transition 56.

CircFAT1 was reported to function as a microRNA sponge in different miRNAs/mRNA regulatory cascades in different systems, although several of the studies were preliminary *in vitro* observations. We are fully aware that the downstream regulatory cascades of a regulator can vary in different biological systems, however functional conservation of regulatory pathways is the mainstream. It is intriguing that all the reported circFAT1 target different miRNA/mRNA. We evaluated all the reported miRNAs and their target mRNAs in our setting, finding that half of reported miRNAs was not expressed in A549 cells; as to those expressed, the RNAs were not regulated or regulated in opposite direction by circFAT1, indicating that tumor cells were highly flexible to evolve different strategies to benefit tumor cell growth in different types of cancers. To add another layer of complexity, while circFAT1 function as an oncogene in most cases, it was downregulated in gastric cancer and function as a tumor suppressor. In contrast to most reports that circFAT1 was present in cytoplasm, it was present in cytoplasm to upregulating tumor suppressor RUNX1 by acting as a sponge for miR-548g, and in nuclei to regulate YBX1 function by physical interaction in gastric cancer 32. Consistent with our finding, circFAT1 was reported to be upregulated in five NSCLC patients tested, although we found that the reported target miR-30e-5p was reduced by 51% and USP22 mRNA did not change significantly in A549 cells upon shcircFAT1 treatment, instead of an increase of miR-30e-5p by about 4 fold and a reduction of USP22 by half in the report 36. This discrepancy could be due to different experimental conditions.

While we demonstrated that miR-7/IRS2/ERK/CCND1 played an important role in mediating circFAT1 function, our data doesn't rule out the possibilities that circFAT1 has other completely different cellular functions, other regulatory pathways, or regulating other untested microRNA/mRNA/protein targets. RNA profiling of A549 cells transfected with miR-7 mimic demonstrated that the expression of 1756 genes were altered (Table S6). It is possible that other genes, directly or indirectly regulated by miR-7, may also play a role in the circFAT1 function. Similarly, IRS2 has been reported to regulates both ERK and PI3K/AKT pathways 54. Although PI3K/AKT pathway plays a very important role in tumor growth, it is also reported to play a role in regulating apoptosis 59, which we didn't observe the phenotype in circFAT1 knockdown cells. Whether PI3K/AKT also plays a role in the regulation of cell proliferation by circFAT1 and, if it does, their downstream targets remain to be determined.

CircRNAs were generally considered to be expressed with high tissue and cell specificity, circFAT1 played a vital role in several types of cancers, including cervical cancer (CC), head and neck squamous cell carcinoma (HNSCC), hepatocellular carcinoma (HCC), osteosarcoma (OS), colorectal cancer (CRC), papillary thyroid cancer (PTC), breast cancer (BC) and NSCLC 27-30, 33, 35-37, which underscore the importance of circFAT1 in tumorigenesis, thus it could be a candidate of pan-cancer markers for cancer screening. In this context, it was essential to establish the relationship between circFAT1 levels, ideally in body fluids, and various cancers at different stages. In this study, we further expanded our observations to therapeutic impact of circFAT1 on chemotherapy, and finding that downregulation of circFAT1 dramatically reduced tumor weight and size in both high and low dosages of DDP. These results suggest that circFAT1 level may partly predict effectiveness of DDP, perhaps other therapies as well, and that tumors with lower circFAT1 may require lower dosage of treatment, which is desirable for patients with lower tolerances to chemotherapy drugs, such as DDP. Detailed analysis of interactions between circFAT1 expression in tumors or body fluids and the effectiveness of other cancer therapies promise for a precision medicine approach to optimize therapy strategies.

**Figure 7:**
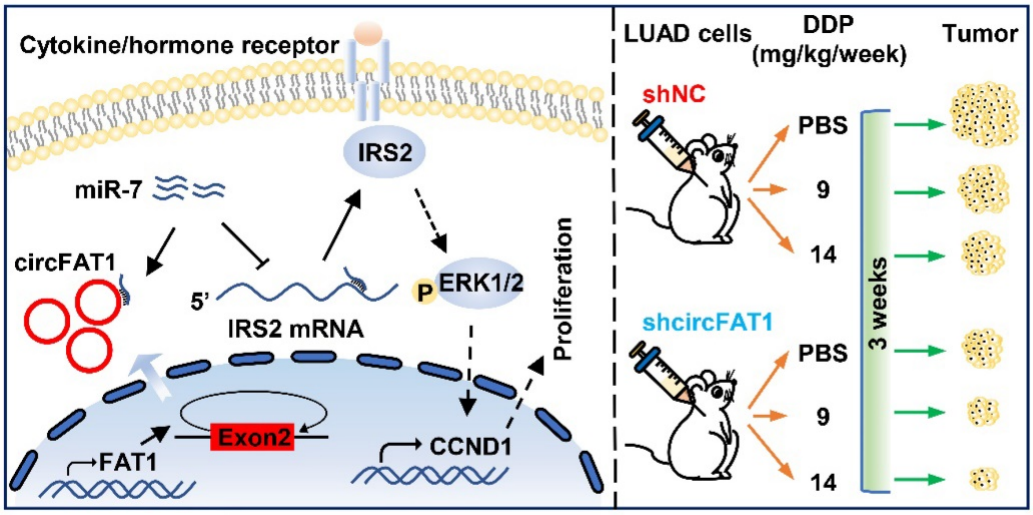
** Working model of circFAT1 action.** (Left) CircFAT1 sequence-specifically sequester miR-7, resulting in an increase of IRS2 mRNA and protein, which in turn enhance ERK1/2 phosphorylation and CCND1 production, consequently promoting tumor cell growth. (Right) CircFAT1 enhances the effectiveness of the chemotherapeutic drug DDP.

In summary, our study demonstrates that circFAT1 promotes tumorigenesis in LUAD through regulating miR-7/IRS2/ERK1/2/CCND1 axis and affects effectiveness of DDP (Figure 7). Our findings shed light on the underlying mechanisms of LUAD tumorigenesis, and suggest that circFAT1 could be a therapeutic target and an important marker for precision treatment of LUAD.

## Supplementary Material

Supplementary figures.

Supplementary tables.

## Abbreviations

NSCLC: non-small cell lung cancer
LUAD: lung adenocarcinoma
LUSC: lung squamous carcinoma
LCLC: large-cell lung cancer
SCLC: small cell lung cancer
EGFR: epidermal growth factor receptor
HER2: erb-b2 receptor tyrosine kinase 2
ALK: anaplastic lymphoma kinase
KRAS: Kirsten viral rat sarcoma (v-RAS) homolog
circRNA: circular ribonucleic acid
miRNA: micro ribonucleic acid
UTR: untranslated region
RISC: RNA-induced silencing complex
IRES: internal ribosome entry sites
ORF: open reading frame
FAT1: FAT atypical cadherin 1
FBS: fetal bovine serum
RT-qPCR: reverse transcription quantitative polymerase chain reaction
GADPH: glyceraldehyde-3-phosphate dehydrogenase
U6: U6 small nuclear 1
MALAT1: metastasis associated lung adenocarcinoma transcript 1
shNC: negative control shRNA
CCK8: Cell Counting Kit 8
BrdU: 5-bromo-2'-deoxyuridine
DAPI: 4',6-diamidino-2-phenylindole
PI: propidium iodide
FITC: fluorescein isothiocyanate
7-AAD: 7-amino-actinomycin D
DEPC: diethyl pyrocarbonate
PBS: phosphate buffered saline
IF: Immunofluorescence
BSA: Bovine serum albumin
PMSF: phenylmethylsulfonyl fluoride
IRS2: insulin receptor substrate 2
CCND1: cyclin D1
ERK: extracellular signal regulated kinase
WT: wildtype
Mut: mutant
DDP: cisplatin
CC: cervical cancer
HNSCC: head and neck squamous cell carcinoma
HCC: hepatocellular carcinoma
OS: osteosarcoma
CRC: colorectal cancer
PTC: papillary thyroid cancer
BC: breast cancer

## References

[1] Sung H, Ferlay J, Siegel RL, Laversanne M, Soerjomataram I, Jemal A (2021). Global Cancer Statistics 2020: GLOBOCAN Estimates of Incidence and Mortality Worldwide for 36 Cancers in 185 Countries. CA Cancer J Clin.

[2] Travis WD, Brambilla E, Riely GJ (2013). New pathologic classification of lung cancer: relevance for clinical practice and clinical trials. J Clin Oncol.

[3] Travis WD, Brambilla E, Nicholson AG, Yatabe Y, Austin JHM, Beasley MB (2015). The 2015 World Health Organization Classification of Lung Tumors: Impact of Genetic, Clinical and Radiologic Advances Since the 2004 Classification. J Thorac Oncol.

[4] Siegel RL, Miller KD, Fuchs HE, Jemal A (2021). Cancer Statistics, 2021. CA Cancer J Clin.

[5] Nicholson AG, Chansky K, Crowley J, Beyruti R, Kubota K, Turrisi A (2016). The International Association for the Study of Lung Cancer Lung Cancer Staging Project: Proposals for the Revision of the Clinical and Pathologic Staging of Small Cell Lung Cancer in the Forthcoming Eighth Edition of the TNM Classification for Lung Cancer. J Thorac Oncol.

[6] Sabari JK, Lok BH, Laird JH, Poirier JT, Rudin CM (2017). Unravelling the biology of SCLC: implications for therapy. Nat Rev Clin Oncol.

[7] Petersen I, Warth A (2016). Lung cancer: developments, concepts, and specific aspects of the new WHO classification. J Cancer Res Clin Oncol.

[8] Lemjabbar-Alaoui H, Hassan OU, Yang YW, Buchanan P (2015). Lung cancer: Biology and treatment options. Biochim Biophys Acta.

[9] Yoneda K, Imanishi N, Ichiki Y, Tanaka F (2019). Treatment of Non-small Cell Lung Cancer with EGFR-mutations. J UOEH.

[10] Shaw AT, Solomon BJ, Besse B, Bauer TM, Lin CC, Soo RA (2019). ALK Resistance Mutations and Efficacy of Lorlatinib in Advanced Anaplastic Lymphoma Kinase-Positive Non-Small-Cell Lung Cancer. J Clin Oncol.

[11] Suzuki H, Zuo Y, Wang J, Zhang MQ, Malhotra A, Mayeda A (2006). Characterization of RNase R-digested cellular RNA source that consists of lariat and circular RNAs from pre-mRNA splicing. Nucleic Acids Res.

[12] Li Y, Zheng Q, Bao C, Li S, Guo W, Zhao J (2015). Circular RNA is enriched and stable in exosomes: a promising biomarker for cancer diagnosis. Cell Res.

[13] Jeck WR, Sorrentino JA, Wang K, Slevin MK, Burd CE, Liu J (2013). Circular RNAs are abundant, conserved, and associated with ALU repeats. RNA.

[14] Huang G, Liang M, Liu H, Huang J, Li P, Wang C (2020). CircRNA hsa_circRNA_104348 promotes hepatocellular carcinoma progression through modulating miR-187-3p/RTKN2 axis and activating Wnt/beta-catenin pathway. Cell Death Dis.

[15] Yang R, Xing L, Zheng X, Sun Y, Wang X, Chen J (2019). The circRNA circAGFG1 acts as a sponge of miR-195-5p to promote triple-negative breast cancer progression through regulating CCNE1 expression. Mol Cancer.

[16] Della Bella E, Menzel U, Basoli V, Tourbier C, Alini M, Stoddart MJ (2020). Differential Regulation of circRNA, miRNA, and piRNA during Early Osteogenic and Chondrogenic Differentiation of Human Mesenchymal Stromal Cells. Cells.

[17] Yu F, Zhang Y, Wang Z, Gong W, Zhang C (2021). Hsa_circ_0030042 regulates abnormal autophagy and protects atherosclerotic plaque stability by targeting eIF4A3. Theranostics.

[18] Hammond SM (2015). An overview of microRNAs. Adv Drug Deliv Rev.

[19] Hansen TB, Jensen TI, Clausen BH, Bramsen JB, Finsen B, Damgaard CK (2013). Natural RNA circles function as efficient microRNA sponges. Nature.

[20] Panda AC (2018). Circular RNAs Act as miRNA Sponges. Adv Exp Med Biol.

[21] Du WW, Yang W, Chen Y, Wu ZK, Foster FS, Yang Z (2017). Foxo3 circular RNA promotes cardiac senescence by modulating multiple factors associated with stress and senescence responses. Eur Heart J.

[22] Du WW, Zhang C, Yang W, Yong T, Awan FM, Yang BB (2017). Identifying and Characterizing circRNA-Protein Interaction. Theranostics.

[23] Li Z, Huang C, Bao C, Chen L, Lin M, Wang X (2015). Exon-intron circular RNAs regulate transcription in the nucleus. Nat Struct Mol Biol.

[24] Yang Y, Fan X, Mao M, Song X, Wu P, Zhang Y (2017). Extensive translation of circular RNAs driven by N(6)-methyladenosine. Cell Res.

[25] Lei M, Zheng G, Ning Q, Zheng J, Dong D (2020). Translation and functional roles of circular RNAs in human cancer. Mol Cancer.

[26] Yang Y, Wang Z (2019). IRES-mediated cap-independent translation, a path leading to hidden proteome. J Mol Cell Biol.

[27] Jia L, Wang Y, Wang CY (2021). circFAT1 Promotes Cancer Stemness and Immune Evasion by Promoting STAT3 Activation. Adv Sci (Weinh).

[28] Liu G, Huang K, Jie Z, Wu Y, Chen J, Chen Z (2018). CircFAT1 sponges miR-375 to promote the expression of Yes-associated protein 1 in osteosarcoma cells. Mol Cancer.

[29] Zhou B, Li T, Xie R, Zhou J, Liu J, Luo Y (2021). CircFAT1 facilitates cervical cancer malignant progression by regulating ERK1/2 and p38 MAPK pathway through miR-409-3p/CDK8 axis. Drug Dev Res.

[30] Wei H, Yan S, Hui Y, Liu Y, Guo H, Li Q (2020). CircFAT1 promotes hepatocellular carcinoma progression via miR-30a-5p/REEP3 pathway. J Cell Mol Med.

[31] Hu B, Xian Z, Zou Q, Zhang D, Su D, Yao J (2021). CircFAT1 Suppresses Colorectal Cancer Development Through Regulating miR-520b/UHRF1 Axis or miR-302c-3p/UHRF1 Axis. Cancer Biother Radiopharm.

[32] Fang J, Hong H, Xue X, Zhu X, Jiang L, Qin M (2019). A novel circular RNA, circFAT1(e2), inhibits gastric cancer progression by targeting miR-548g in the cytoplasm and interacting with YBX1 in the nucleus. Cancer Lett.

[33] Yao Y, Li X, Cheng L, Wu X, Wu B (2021). Circular RNA FAT atypical cadherin 1 (circFAT1)/microRNA-525-5p/spindle and kinetochore-associated complex subunit 1 (SKA1) axis regulates oxaliplatin resistance in breast cancer by activating the notch and Wnt signaling pathway. Bioengineered.

[34] Gu H, Cheng X, Xu J, Zhou K, Bian C, Chen G (2020). Circular RNA circFAT1(e2) Promotes Osteosarcoma Progression and Metastasis by Sponging miR-181b and Regulating HK2 Expression. Biomed Res Int.

[35] Pan F, Zhang D, Li N, Liu M (2021). Circular RNA circFAT1(e2) Promotes Colorectal Cancer Tumorigenesis via the miR-30e-5p/ITGA6 Axis. Comput Math Methods Med.

[36] Dong W, Zhang H, Dai Y, Zhou Y, Luo Y, Zhao C (2021). circRNA circFAT1(e2) Elevates the Development of Non-Small-Cell Lung Cancer by Regulating miR-30e-5p and USP22. Biomed Res Int.

[37] Liu J, Li H, Wei C, Ding J, Lu J, Pan G (2020). circFAT1(e2) Promotes Papillary Thyroid Cancer Proliferation, Migration, and Invasion via the miRNA-873/ZEB1 Axis. Comput Math Methods Med.

[38] Liu M (2021). CircFAT1 is Overexpressed in Colorectal Cancer and Suppresses Cancer Cell Proliferation, Invasion and Migration by Increasing the Maturation of miR-10a. Cancer Manag Res.

[39] Wu W, Zhou J, Wu Y, Tang X, Zhu W (2021). Overexpression of circRNA circFAT1 in Endometrial Cancer Cells Increases Their Stemness by Upregulating miR-21 Through Methylation. Cancer Biother Radiopharm.

[40] Varas-Godoy M, Lladser A, Farfan N, Villota C, Villegas J, Tapia JC (2018). *In vivo* knockdown of antisense non-coding mitochondrial RNAs by a lentiviral-encoded shRNA inhibits melanoma tumor growth and lung colonization. Pigment Cell Melanoma Res.

[41] Hsiao KY, Lin YC, Gupta SK, Chang N, Yen L, Sun HS (2017). Noncoding Effects of Circular RNA CCDC66 Promote Colon Cancer Growth and Metastasis. Cancer Res.

[42] Evilsizor MN, Ray-Jones HF, Lifshitz J, Ziebell J Primer for immunohistochemistry on cryosectioned rat brain tissue: example staining for microglia and neurons. J Vis Exp. 2015: e52293.

[43] Torres M, Becquet D, Guillen S, Boyer B, Moreno M, Blanchard MP RNA Pull-down Procedure to Identify RNA Targets of a Long Non-coding RNA. J Vis Exp. 2018: 57379.

[44] Gao Y, Wang J, Zhao F (2015). CIRI: an efficient and unbiased algorithm for de novo circular RNA identification. Genome Biol.

[45] Memczak S, Jens M, Elefsinioti A, Torti F, Krueger J, Rybak A (2013). Circular RNAs are a large class of animal RNAs with regulatory potency. Nature.

[46] Krzywinski M, Schein J, Birol I, Connors J, Gascoyne R, Horsman D (2009). Circos: an information aesthetic for comparative genomics. Genome Res.

[47] Dudekula DB, Panda AC, Grammatikakis I, De S, Abdelmohsen K, Gorospe M (2016). CircInteractome: A web tool for exploring circular RNAs and their interacting proteins and microRNAs. RNA Biol.

[48] Korac P, Antica M, Matulic M (2021). MiR-7 in Cancer Development. Biomedicines.

[49] Li JH, Liu S, Zhou H, Qu LH, Yang JH (2014). starBase v2.0: decoding miRNA-ceRNA, miRNA-ncRNA and protein-RNA interaction networks from large-scale CLIP-Seq data. Nucleic Acids Res.

[50] Liu W, Wang X (2019). Prediction of functional microRNA targets by integrative modeling of microRNA binding and target expression data. Genome Biol.

[51] Chen Y, Wang X (2020). miRDB: an online database for prediction of functional microRNA targets. Nucleic Acids Res.

[52] Zhang P, Shao G, Lin X, Liu Y, Yang Z (2017). MiR-338-3p inhibits the growth and invasion of non-small cell lung cancer cells by targeting IRS2. Am J Cancer Res.

[53] Yang Z, Lin X, Zhang P, Liu Y, Liu Z, Qian B (2020). Long non-coding RNA LINC00525 promotes the non-small cell lung cancer progression by targeting miR-338-3p/IRS2 axis. Biomed Pharmacother.

[54] Machado-Neto JA, Fenerich BA, Rodrigues Alves APN, Fernandes JC, Scopim-Ribeiro R, Coelho-Silva JL (2018). Insulin Substrate Receptor (IRS) proteins in normal and malignant hematopoiesis. Clinics (Sao Paulo).

[55] Gao F, Li M, Zhou L, Liu W, Zuo H, Li W (2020). Xanthohumol targets the ERK1/2Fra1 signaling axis to reduce cyclin D1 expression and inhibit nonsmall cell lung cancer. Oncol Rep.

[56] Wikman H, Kettunen E (2006). Regulation of the G1/S phase of the cell cycle and alterations in the RB pathway in human lung cancer. Expert Rev Anticancer Ther.

[57] Wang S, Zhang K, Tan S, Xin J, Yuan Q, Xu H (2021). Circular RNAs in body fluids as cancer biomarkers: the new frontier of liquid biopsies. Mol Cancer.

[58] Li J, Sun D, Pu W, Wang J, Peng Y (2020). Circular RNAs in Cancer: Biogenesis, Function, and Clinical Significance. Trends Cancer.

[59] Noorolyai S, Shajari N, Baghbani E, Sadreddini S, Baradaran B (2019). The relation between PI3K/AKT signalling pathway and cancer. Gene.

