# Supplementary material for: CircFAT1 Promotes Lung Adenocarcinoma Progression by Sequestering miR-7 from Repressing IRS2-ERK-mediated CCND1 Expression
Source: Int J Biol Sci. 2022 Jun 13;18(10):3944–60. doi: 10.7150/ijbs.70889 (PMC9274483; doi:10.7150/ijbs.70889)
Supplement: Supplementary file 1 — Supplementary figures. [file ijbsv18p3944s1.pdf]

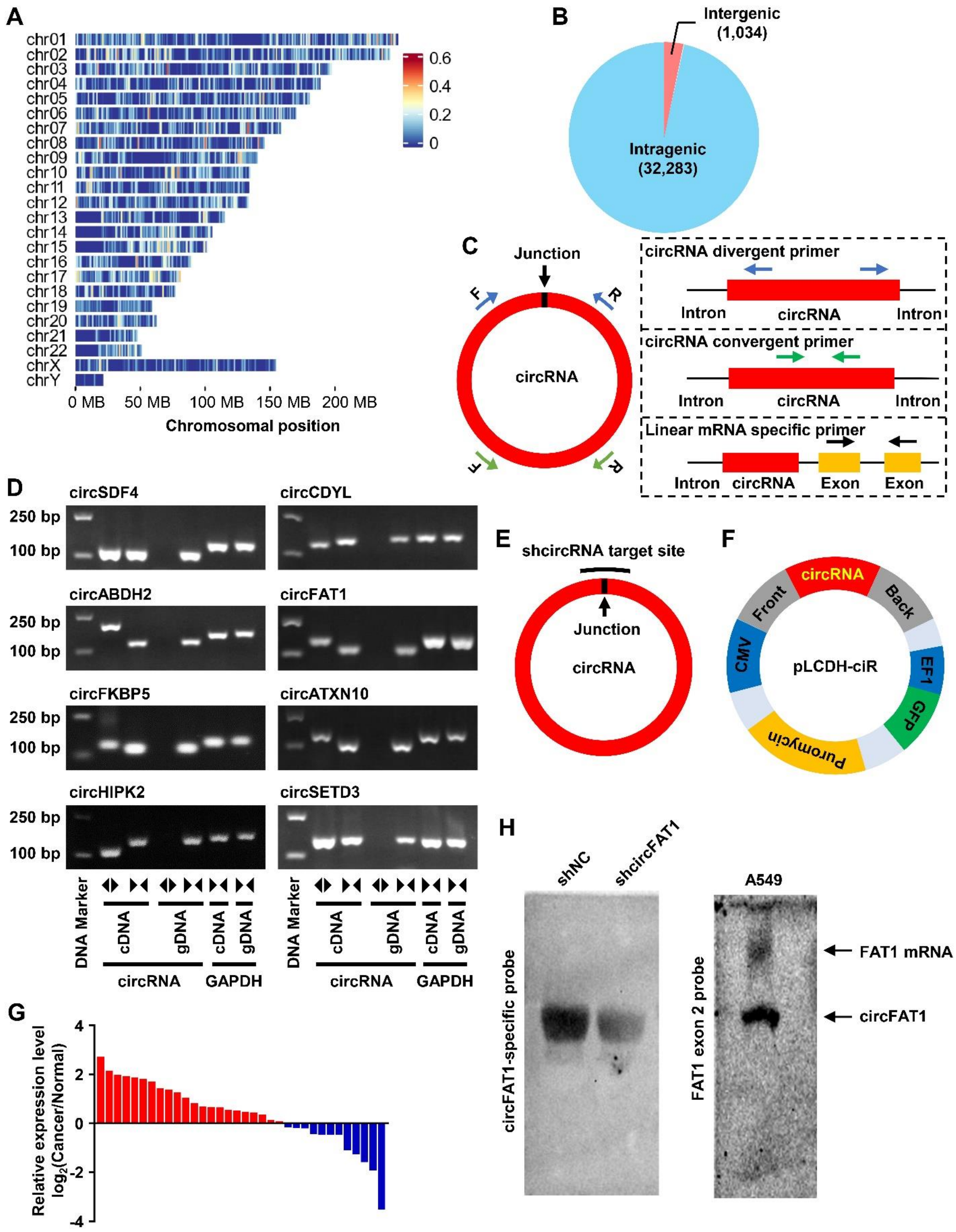
A
chr01
chr02
chr03
chr04
chr05
chr06
chr07
chr08
chr09
chr10
chr11
chr12
chr13
chr14
chr15
chr16
chr17
chr18
chr19
chr20
chr21
chr22
chrX
chrY
0.6
0.4
0.2
0
0 MB
50 MB
100 MB
150 MB
200 MB
Chromosomal position
B
Intergenic (1,034)
Intragenic (32,283)
C
Junction
F
R
circRNA
circRNA divergent primer
Intron
circRNA
Intron
circRNA convergent primer
Intron
circRNA
Intron
Linear mRNA specific primer
Intron
circRNA
Exon
Exon
D
circSDF4
circCDYL
circABDH2
circFAT1
circFKBP5
circATXN10
circHIPK2
circSETD3
250 bp
100 bp
DNA Marker
cDNA
gDNA
cDNA
gDNA
circRNA
GAPDH
E
shcircRNA target site
Junction
circRNA
F
circRNA
Front
Back
CMV
pLCDH-ciR
EF1
GFP
Puromycin
G
Relative expression level
log2(Cancer/Normal)
4
2
0
-2
-4
H
shNC
shcircFAT1
circFAT1-specific probe
A549
FAT1 exon 2 probe
FAT1 mRNA
circFAT1

Figure S1. Genomic distribution, RT-PCR validation of CircRNAs identified in human A549 cells and circFAT1 expression in LUAD tumors. **(A)** The chromosomal distribution of circRNAs identified in A549 cells. **(B)** Pie chart showing the numbers of intragenic and intergenic circRNAs identified in A549 cells. **(C)** The schematic illustration of divergent primers, convergent primers of circRNA, and linear mRNA-specific primers designed from linear RNA-specific exons. **(D)** CircRNAs were validated by RT-PCR, and the PCR products were separated by gel electrophoresis. cDNA but not gDNA were amplified by divergent primers specific for circRNAs, and both cDNA and gDNA were amplified by convergent primers. GAPDH was used as a linear RNA control. **(E** and **F)** Schematic illustration of shcircRNA target site (E) and the map of the circRNA overexpression vector pLCDH-ciR (F). **(G)** Bar graph showing relative changes of circFAT1 in every sample pair displayed in Figure 1I. **(H)** The existence of circFAT1 was confirmed with Northern blotting by using a circFAT1-specific probe and a FAT1 exon 2 probe. F, forward primer; R, reverse primer.

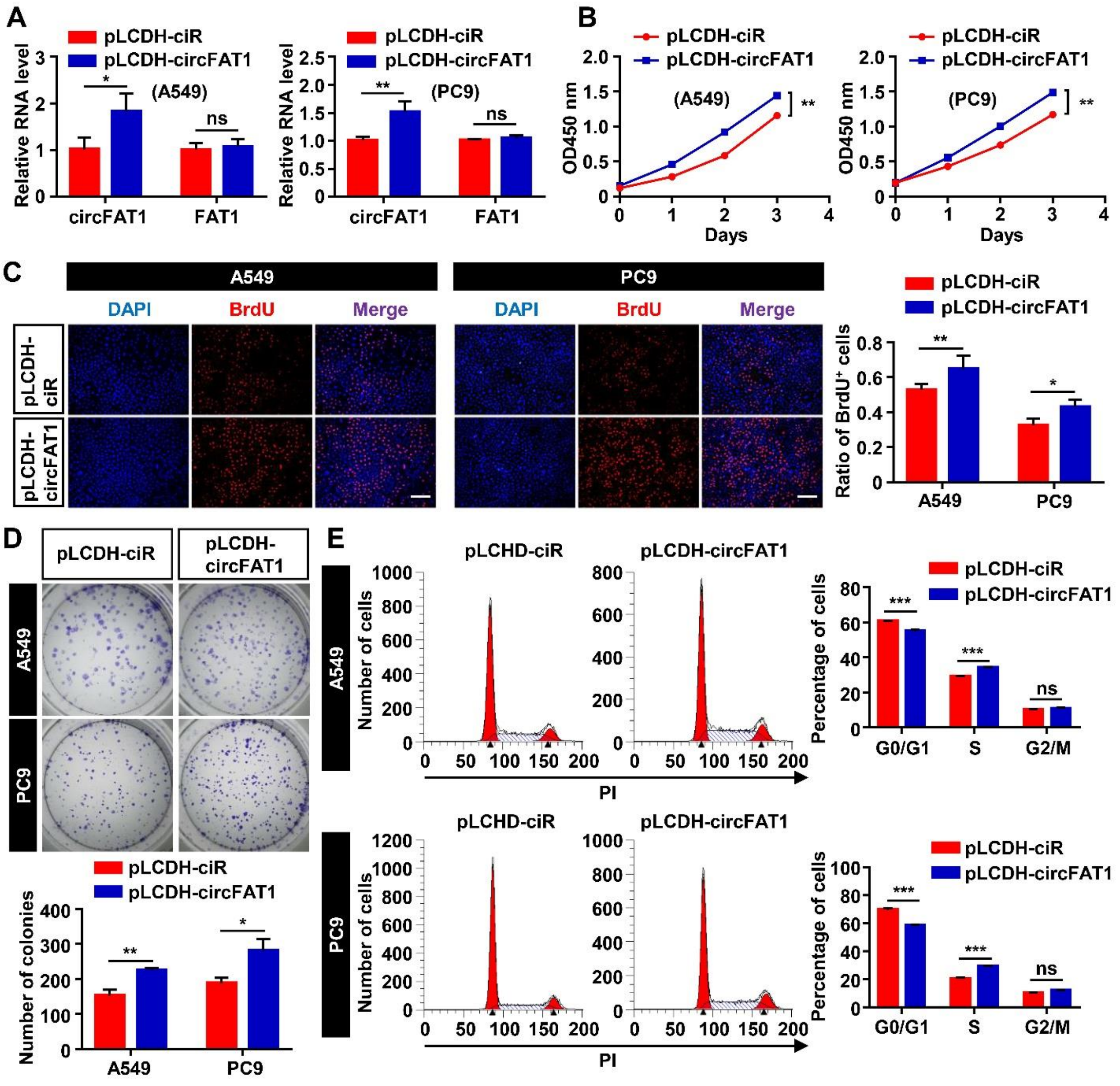

Figure S2. CircFAT1 promotes A549 and PC9 cells proliferation (gain-of-function assays**). (A)** RT-qPCR analysis confirmed circFAT1 overexpression without altering linear FAT1 mRNA expression. **(B)** CCK8 assay demonstrated that overexpression of circFAT1 promoted LUAD cell proliferation. **(C,** left**)** BrdU assay showing the enhancement of BrdU incorporation by circFAT1. (Right) Quantification of BrdU labeled cells. **(D,** Upper**)** Colony formation assay to assess clonogenicity of circFAT1-overexpressing cells. (Bottom) Quantification of colonies with more than 50 cells. **(E)** Overexpression of circFAT1 reduced the cells at G0/G1 phase and increased the cells at S phase. Data are presented as mean ± SD; *$P < 0.05$, **$P < 0.01$, ***$P < 0.001$; ns, not significant.

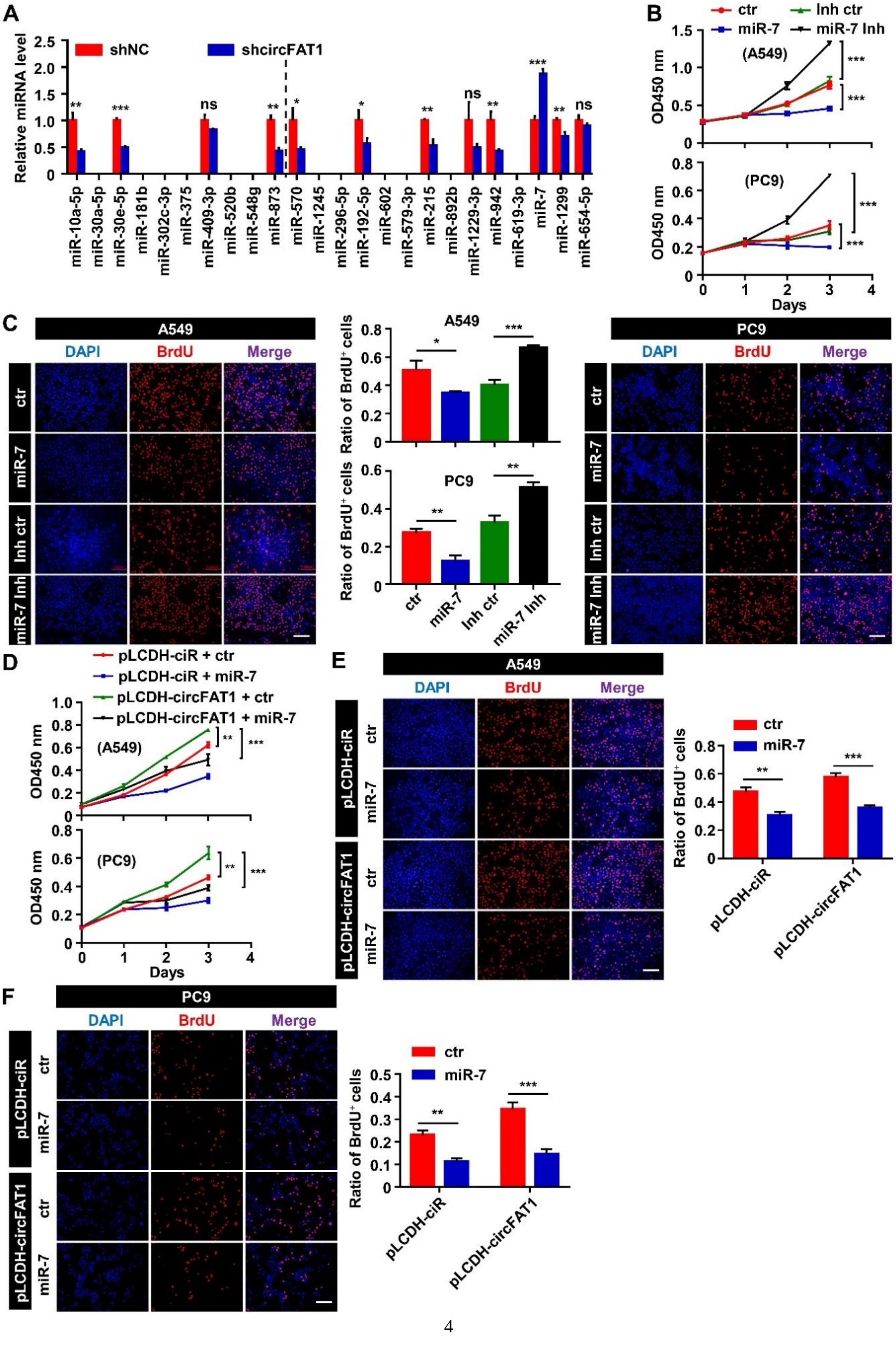

A
Relative miRNA level
shNC
shcircFAT1
miR-10a-5p
miR-30a-5p
miR-30e-5p
miR-181b
miR-302c-3p
miR-375
miR-409-3p
miR-520b
miR-548g
miR-873
miR-570
miR-1245
miR-296-5p
miR-192-5p
miR-602
miR-579-3p
miR-215
miR-892b
miR-1229-3p
miR-942
miR-619-3p
miR-7
miR-1299
miR-654-5p
B
ctr
Inh ctr
miR-7
miR-7 Inh
OD450 nm
(A549)
(PC9)
Days
C
A549
PC9
DAPI
BrdU
Merge
ctr
miR-7
Inh ctr
miR-7 Inh
Ratio of BrdU⁺ cells
D
pLCDH-ciR + ctr
pLCDH-ciR + miR-7
pLCDH-circFAT1 + ctr
pLCDH-circFAT1 + miR-7
OD450 nm
(A549)
(PC9)
Days
E
A549
DAPI
BrdU
Merge
pLCDH-ciR
pLCDH-circFAT1
ctr
miR-7
Ratio of BrdU⁺ cells
F
PC9
DAPI
BrdU
Merge
pLCDH-ciR
pLCDH-circFAT1
ctr
miR-7
Ratio of BrdU⁺ cells

Figure S3. MiR-7 inhibits LUAD cell proliferation and rescues circFAT1 overexpression phenotype. (A) Expression levels of predicted or reported miRNAs targeted by circFAT1 in shcircFAT1- or shNC-expressing A549 cells. MiR-7 was upregulated in circFAT1 knockdown cells. **(B)** CCK8 assay showing growth curves of A549 and PC9 cells transfected with the miR-7 mimic or the miR-7 inhibitor; MiR-7 inhibited LUAD cell growth. **(C)** BrdU assay in A549 (Left) and PC9 (Right) cells transfected with the miR-7 mimic or the miR-7 inhibitor, showing inhibition of cell proliferation by miR-7. (Middle) Quantification of BrdU-incorporated cells. **(D-F)** CCK-8 (D: Upper, A549; Lower, PC9) and BrdU (E, A549 cells; F, PC9 cells) assays showing miR-7 capable of rescuing circFAT1 overexpression phenotype in A549 and PC9 cells. Data are presented as mean ± SD; *$P < 0.05$, **$P < 0.01$, ***$P < 0.001$; ns, not significant; ctr, miR-7 mimic control; Inh, miR-7 inhibitor; Inh ctr, miR-7 inhibitor control.

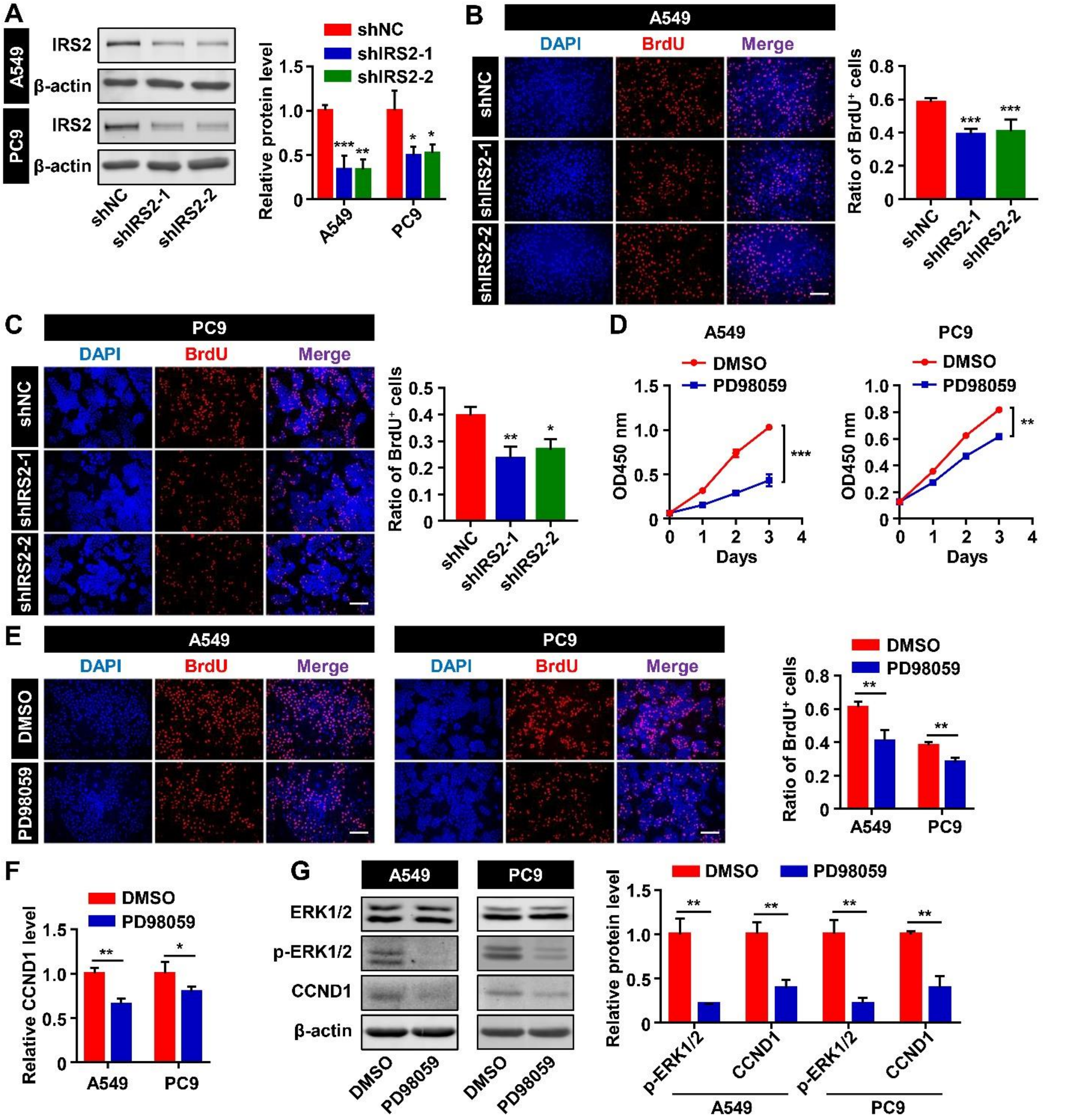

Figure S4. IRS2 and ERK1/2 promote LUAD cell proliferation and increase CCND1 mRNA and protein levels. (A, Left**)** Western blot showing knockdown efficiency of IRS2 at protein levels in A549 and PC9 cells. (Right). Quantification of the relative IRS2 protein levels in the left panel. **(B** and **C,** Left**)** BrdU assays showing shIRS2 inhibited cell proliferation in A549 (B) and PC9 (C). (Right) Quantification of BrdU-incorporated cells. Scale bar, 200 μm. **(D)** CCK8 assay showing growth curves of PD98059-treated A549 (Left) and PC9 (Right) cells. **(E)** BrdU assay showing that the downregulation of ERK1/2 phosphorylation levels by the upstream kinase inhibitor PD98059 inhibited A549 (Left) and PC9 (Middle) cell proliferation; (Right) Quantification of cells in the left and middle panels. **(F** and **G)** PD98059 reduced CCND1 mRNA expression detected by RT-qPCR (F) and p-ERK1/2 and CCND1 protein levels detected by western blot (G, Left) in A549 and PC9 cells. (G, Right)

Quantification of signals in the left panels. The antibodies for ERK and p-ERK detected both ERK1 and ERK2. Data are presented as mean ± SD; *$P < 0.05$, **$P < 0.01$, ***$P < 0.001$; ns, not significant.

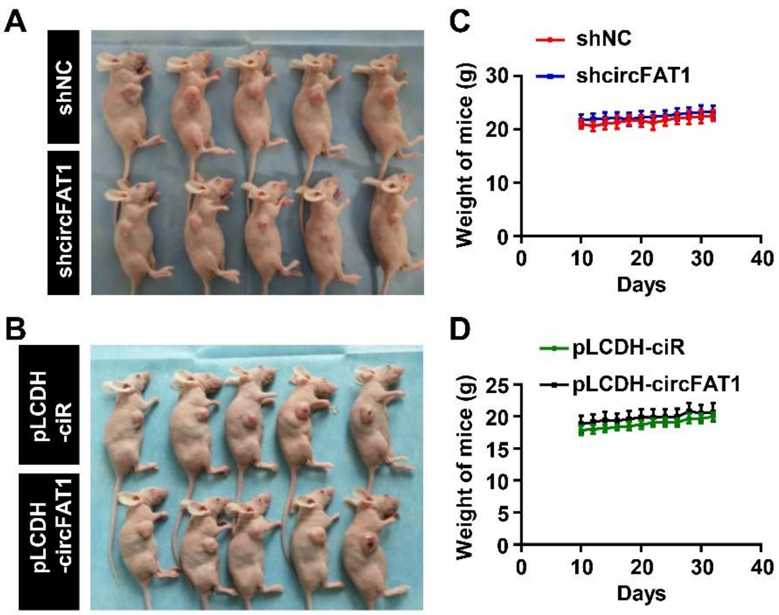

Figure S5. The whole animal view and body weight of xenografted mice. (A and **B)** Secrificed mice bearing xenograft tumors derived from A549 cells with circFAT1 knockdown (A) or overexpression (B) (n=5). **(C** and **D)** Average body weight of mice showed in (A) and (B).

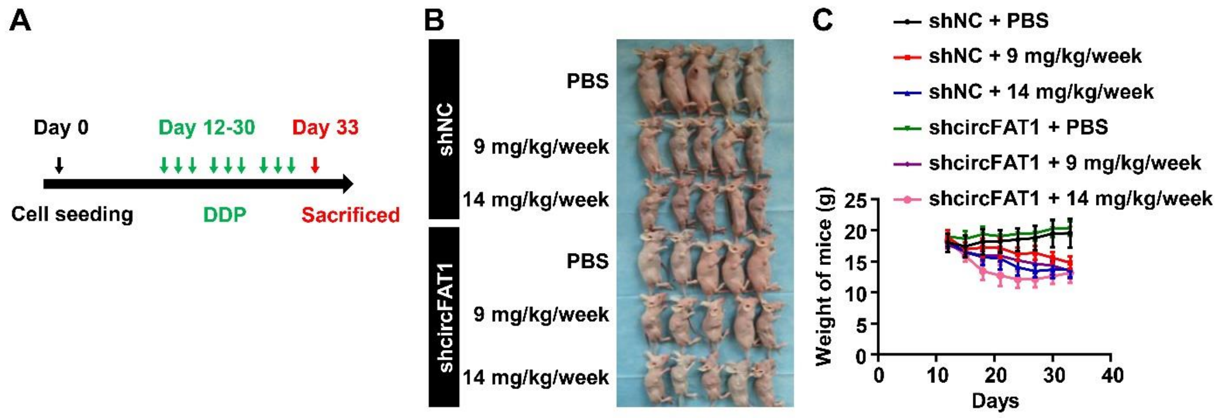

Figure S6. The whole animal view and body weight of xenografted mice bearing tumors derived from circFAT1-downregulated A549 cells and/or treated with different dosages of DDP. (A) Schematic illustration of cell seeding and drug delivery schedule. **(B)** Secrificed mice with xenograft tumors of different treatments as indicated (n=5). **(C)** Body weight of mice shown in (B).
